# Regulation of fruit ascorbic acid concentrations during ripening in high and low vitamin C tomato cultivars

**DOI:** 10.1186/1471-2229-12-239

**Published:** 2012-12-17

**Authors:** Ifigeneia Mellidou, Johan Keulemans, Angelos K Kanellis, Mark W Davey

**Affiliations:** 1 Laboratory for Fruit Breeding and Biotechnology, Department of Biosystems, Faculty of Bioscience Engineering, Katholieke Universiteit Leuven, B-3001, Heverlee, Belgium; 2 Group of Biotechnology of Pharmaceutical Plants. Laboratory of Pharmacognosy, Department of Pharmaceutical Sciences, Aristotle University of Thessaloniki, 541 24, Thessaloniki, Greece

**Keywords:** Ascorbate biosynthesis, Ascorbate recycling, Ascorbate turnover, Candidate gene, GDP-L-galactose phosphorylase, Gene expression, Monodehydroascorbate reductase

## Abstract

**Background:**

To gain insight into the regulation of fruit ascorbic acid (AsA) pool in tomatoes, a combination of metabolite analyses, non-labelled and radiolabelled substrate feeding experiments, enzyme activity measurements and gene expression studies were carried out in fruits of the ‘low-’ and ‘high-AsA’ tomato cultivars ‘Ailsa Craig’ and ‘Santorini’ respectively.

**Results:**

The two cultivars exhibited different profiles of total AsA (totAsA, AsA + dehydroascorbate) and AsA accumulation during ripening, but both displayed a characteristic peak in concentrations at the breaker stage. Substrate feeding experiments demonstrated that the L-galactose pathway is the main AsA biosynthetic route in tomato fruits, but that substrates from alternative pathways can increase the AsA pool at specific developmental stages. In addition, we show that young fruits display a higher AsA biosynthetic capacity than mature ones, but this does not lead to higher AsA concentrations due to either enhanced rates of AsA breakdown (‘Ailsa Craig’) or decreased rates of AsA recycling (‘Santorini’), depending on the cultivar. In the later stages of ripening, differences in fruit totAsA-AsA concentrations of the two cultivars can be explained by differences in the rate of AsA recycling activities. Analysis of the expression of AsA metabolic genes showed that only the expression of one orthologue of GDP-L-galactose phosphorylase (*SlGGP1*), and of two monodehydroascorbate reductases (*SlMDHAR1* and *SlMDHAR3*) correlated with the changes in fruit totAsA-AsA concentrations during fruit ripening in ‘Ailsa Craig’, and that only the expression of *SlGGP1* was linked to the high AsA concentrations found in red ripe ‘Santorini’ fruits.

**Conclusions:**

Results indicate that ‘Ailsa Craig’ and ‘Santorini’ use complementary mechanisms to maintain the fruit AsA pool. In the low-AsA cultivar (‘Ailsa Craig’), alternative routes of AsA biosynthesis may supplement biosynthesis via L-galactose, while in the high-AsA cultivar (‘Santorini’), enhanced AsA recycling activities appear to be responsible for AsA accumulation in the later stages of ripening. Gene expression studies indicate that expression of *SlGGP1* and two orthologues of *SlMDHAR* are closely correlated with totAsA-AsA concentrations during ripening and are potentially good candidates for marker development for breeding and selection.

## Background

L-ascorbic acid (AsA, vitamin C) is one of the most abundant low molecular weight antioxidants of plants, exerting a crucial role in the detoxification of reactive oxygen species generated following exposure to (a)biotic stress factors. In addition to its antioxidant properties, AsA also has major roles in plant development and hormone signalling, cell cycle, cell expansion, as a part of the cellular redox system, and as a cofactor for several important enzymes [1,2]. An enhanced fruit AsA pool has also been suggested to be associated with improved postharvest fruit quality in hard fruit species, such as pear and apple [3,4], as well as with higher polyphenolic compounds levels in both apple [4] and tomato [5] fruit. An increased dietary intake of AsA has also long been associated with a decreased incidence of several human stress-related diseases and disorders [6]. Given its importance for all metabolically active tissues, understanding the regulation of plant AsA metabolism has been a topic of widespread interest particularly towards the long-term aim of developing novel AsA-rich cultivars with higher nutritional value, and potentially improved stress-resistance capabilities.

**Figure 1:**
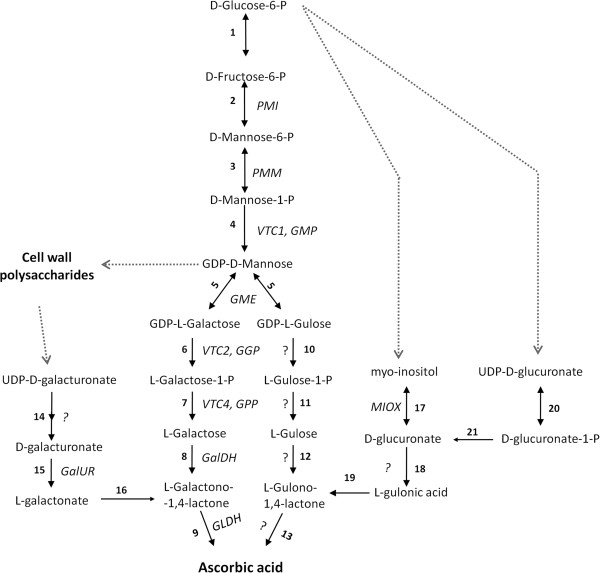
**The main and the alternative L-ascorbic acid biosynthetic pathways in higher plants.** Reactions with question marks yet to be identified. 1, glucose-6-phosphate isomerase; 2, mannose-6-phosphate isomerase (PMI; EC 5.3.1.8); 3, phosphomannomutase (PMM; EC 5.4.2.8); 4, GDP-D-mannose pyrophosphorylase (VTC1 or GMP; EC 2.7.7.13); 5, GDP-D-mannose 3^′^,5^′^-epimerase (GME; EC 5.1.3.18); 6, GDP-L-galactose-phosphorylase (VTC2 or GGP; EC 2.7.7.69); 7, L-galactose-1-P phosphatase (VTC4 or GPP; EC 3.1.3.25); 8, L-galactose dehydrogenase (GalDH; EC 1.1.1.48); 9, L-galactono-1,4-lactone dehydrogenase (GLDH; EC 1.3.2.3); 10, nucleotide pyrophosphatase or sugar-1-P guanyltransferase; 11, sugar phosphatase; 12, sugar dehydrogenase; 13, L-gulono-1,4-lactone oxidase (EC 1.1.3.8); 14, D-galacturonate-1-phosphate uridyltransferase and D-galacturonate-1-phosphate phosphatase (possible); 15, D-galacturonate reductase (GalUR; EC 1.1.1.n9); 16, aldonolactonase; 17, *myo*-inositol oxygenase (MIOX; EC 1.13.99.1); 18, D-glucuronate reductase (EC 1.1.1.19); 19, L-gulonolactonase; 20, D-glucuronate-1-phosphate uridyltransferase; 21, D-glucurono-1-phosphate phosphatase.

In plants, AsA biosynthesis via the L-galactose pathway (Figure 1) as initially described by Wheeler et al. [7] has been proposed to be the main route for AsA accumulation in various species [8-10], and several structural genes from this pathway have been proposed to be key regulators of fruit AsA concentrations in various species. These include GDP-L-galactose phosphorylase (*GGP, VTC2*) in kiwifruit [9] and apple [10], and L-galactose-1-phosphate-phosphatase (*GPP, VTC4*) [11] and GDP-mannose-3,5-epimerase (*GME*) [12] in tomato. Possible alternative biosynthetic pathways (Figure 1) involving uronic acids [13,14], L-gulose [15], or *myo*-inositol [16] have also been demonstrated in several plant species, but to date, there is no convincing evidence that these contribute significantly to the AsA pool under physiological conditions, except possibly in strawberry [14,17]. Nonetheless, we cannot exclude the possibility that these alternative routes could serve to supplement synthesis via L-galactose at certain developmental stages, such as during the later stages of fruit ripening [18].

Oxidised AsA is regenerated, or recycled via the ascorbate-glutathione cycle using reduced glutathione (GSH) as a source of reductant [19]. The primary oxidation product, monodehydroascorbate (MDHA) can spontaneously disproportionate to dehydroascorbate (DHA) and AsA, or be reduced back to AsA via the activity of the NAD(P)-dependent monodehydroascorbate reductase (MDHAR). DHA itself can also be recycled back to AsA via the activity of dehydroascorbate reductase (DHAR). Several studies have demonstrated the importance of the AsA-GSH cycle in preventing the depletion of the AsA pool including in tobacco leaves [20,21], and tomato fruit [22,23]. More recently, overexpression of an orthologue of *DHAR* (GenBank ID: AY971873.1) in tomato (*Solanum lycopersicum*) resulted in an up to 1.6-fold increase in AsA concentrations in mature green and red fruits [24]. However, the same group showed that overexpression of an orthologue of *MDHAR* (GenBank ID: L41345.1) in tomato resulted in 0.7-fold decrease in AsA concentrations in mature green fruits, highlighting the complexity of the control of AsA pool size via recycling.

Tomato has only moderate AsA levels compared to some other fruit species [1], but its importance in the human diet and its high levels of consumption mean that even a relatively small increase in AsA contents can have far reaching consequences for the consumer. We are not aware of a widespread systematic survey of AsA concentrations in tomato cultivars, but data indicates that fruit AsA concentrations of commercial cultivars are significantly lower than those of wild accessions [22], and further show only a limited variation [22,25]. This suggests that there could have been an inadvertent selection for lower AsA contents during the tomato domestication/breeding process.

While several studies on the control of fruit AsA homeostasis in tomatoes are available [11,12,18,22,23,25,26], results to date have been contradictory, possibly due to the use of different cultivars, growth conditions and methodologies. Here, we set out to identify the mechanisms underlying the regulation of tomato fruit total AsA (totAsA) and AsA concentrations during ripening, and to understand why some cultivars are able to accumulate more AsA than others. The experimental approach adopted involves a combination of fruit metabolite analyses, non- and radiolabelled substrate feeding experiments, AsA-recycling and antioxidant enzyme activity measurements, and AsA candidate gene expression profiling throughout ripening. The two cultivars studied were the ‘low-AsA’ ‘Ailsa Craig’, a widely studied model cultivar, and the ‘high-AsA’ ‘Santorini’, a drought-tolerant cultivar originating from the island of ‘Santorini’ in Greece. Integration of all results allowed us to build up a model for regulation in the two cultivars and to identify key regulatory components.

## Results

### Fruit ripening

Under our standardised greenhouse conditions in a hydroponic system, ‘Santorini’ fruit reached maturity slightly faster [50 days after pollination (DAP)], than fruit of ‘Ailsa Craig’ (52DAP). The size of ‘Santorini’ fruit was also on average 60% of the size of ‘Ailsa Craig’ fruit, but there were no significant differences between the cultivars in fruit water contents or total soluble solids contents throughout ripening (Additional file 1: Table S1). To compensate for the differences in ripening time, and to mitigate the influence of variations in environmental conditions over the course of the experiment, fruits from both cultivars were harvested at identical physiological stages throughout the course of the experiment, based on both external and an internal fruit inspection as described in ‘methods’. These fruits were then pooled according to ripening status for analysis.

### Changes in AsA and GSH concentrations during ripening

**Figure 2:**
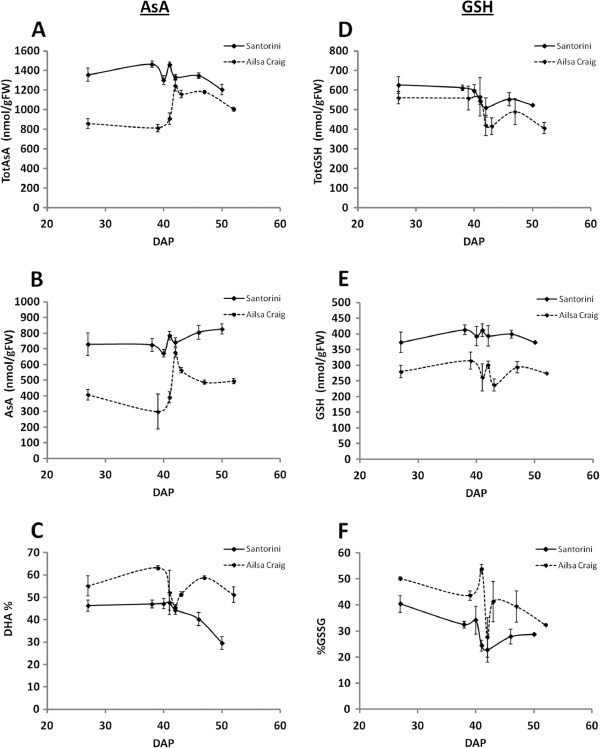
**Determination of L-ascorbic acid and glutathione concentrations during fruit ripening.** Changes in total ascorbic acid (totAsA; **A**), L-ascorbic acid (AsA; **B**), % dehydroascorbate (% DHA; **C**), total glutathione (totGSH; **D**), glutathione (GSH; **E**) and % oxidized glutathione (% GSSG; **F**) of pericarp during fruit ripening of ‘Santorini’ (black lines) and ‘Ailsa Craig’ (dashed lines) fruits grown in a hydroponic system in the greenhouse. Ripening stages are expressed as Days After Pollination (DAP). For ‘Santorini’: 27DAP, immature green (IG); 38DAP, mature green (MG); 40DAP, B-1; 41DAP, breaker (B); 42DAP, B + 1; 46DAP, pink; 50DAP, red. For ‘Ailsa Craig’: 27DAP, IG; 39DAP, MG; 41DAP, B-1; 42DAP, B; 43DAP, B + 1; 47DAP, pink; 52DAP, red. Results are represented as the mean of three biological replications ± SD. Statistical analysis is provided in Additional file 2: Table S2.

The concentrations of totAsA (AsA + DHA), AsA and % DHA of pericarp tissues of the fruit differed significantly between the two cultivars, but also varied during ripening (Figure 2; Additional file 2: Table S2). Specifically, totAsA and AsA concentrations of ‘Santorini’ fruits were on average 1.3-fold and 1.6-fold higher than those of ‘Ailsa Craig’ fruits at the same developmental stage (Figure 2A-B). The largest differences were observed at the MG stage when totAsA and AsA concentrations were 1.8- and 2.4-fold higher in ‘Santorini’ fruits, respectively. This stage represents the end of the cell expansion period, when fruits have reached their final size. At the fully ripe red stage, ‘Santorini’ fruits contained 1.7-fold higher AsA levels than ‘Ailsa Craig’ fruits, but due to a lower proportion of DHA (% DHA; Figure 2C), totAsA levels were only 1.2-fold higher.

The changes in totAsA during ripening showed that totAsA concentrations were high early in development in ‘Santorini’ fruits, but thereafter varied little. In comparison, totAsA concentrations in ‘Ailsa Craig’ fruits were relatively low early in development but increased substantially after the breaker stage. In both cultivars, the highest totAsA concentrations were measured at the breaker stage, where we observed a sharp and short-term spike in concentrations (Figure 2A). To our knowledge, this is the first time that such short-time fluctuations in totAsA concentrations have been observed in tomato or other fruits. Since fruit at each ripening stage were harvested over a period of several weeks and then pooled, these changes in metabolite contents are unrelated to environmental conditions. The changes in fruit AsA concentrations generally followed the totAsA concentration profiles, the only exceptions being a slight increase in the size of the AsA pool size in ‘Santorini’ after the B + 1 stage, and larger concentration changes around the breaker stage in ‘Ailsa Craig (Figure 2B; Additional file 2: Table S2). It is unclear what the biological basis of this spike in totAsA-AsA concentrations at the breaker stage is, but we considered it to be an important diagnostic feature and in this work it was used to help identify candidate genes whose expression was regulated or correlated with changes in fruit totAsA-AsA concentrations.

The proportion of DHA present in the totAsA pool (% DHA) is an indication of the degree of oxidative stress being experienced by the tissue. In ‘Santorini’ fruits, % DHA decreased from 47.7% to 29.6%, during the breaker - red stage transition (Figure 2C), indicating either a decreased level of oxidative stress or an improved capacity for AsA recycling after the onset of ripening. In contrast, the % DHA was consistently higher throughout ripening in ‘Ailsa Craig’ fruits, and at the red stage represented 51.2% of the totAsA pool, compared to only 29.6% in ‘Santorini’ fruits at the same stage. Interestingly, % DHA in ‘Ailsa Craig’ fruits specifically decreased at the breaker stage, but there were no significant changes in ‘Santorini’ fruits (Figure 2C).

The concentrations of total GSH [totGSH; GSH + oxidized GSH (GSSG)], GSH, and % GSSG differed between the two cultivars, and also varied during ripening (Figure 2D-F; Additional file 2: Table S2). Fruit totGSH-GSH concentrations were on average 1.2- and 1.4-fold higher in ‘Santorini’ throughout ripening, with the largest differences being observed at the red stage, where totGSH and GSH concentrations were 1.3 and 1.4-fold higher than in ‘Ailsa Craig’ respectively (Figure 2D-E).

Changes in totGSH-GSH concentrations were similar in both cultivars throughout ripening (Figure 2D-E), again with characteristic, but statistically non-significant, changes in GSH concentrations occurring around the breaker stage (Figure 2E). Highest GSH concentrations were measured at the IG stage in both cultivars, and similar to the results observed for totAsA-AsA pool, the GSH pool was consistently more oxidized in ‘Ailsa Craig’ fruits throughout ripening compared to ‘Santorini’ (Figure 2C and 2F).

### Changes in AsA metabolism during fruit ripening

Substrate feeding experiments with non- and radiolabelled biosynthetic precursors were used to examine changes in the capacity of fruit tissues to take up AsA and DHA, as well as their capacity to synthesise AsA. These experiments had no significant effect on the redox status of the AsA pool of the fruit tissues so that only the changes in tissue totAsA concentrations are presented and discussed. Since we were unable to detect AsA-totAsA in the control incubation medium (results not shown), it is unlikely that a significant diffusion of AsA or DHA from the tissue occurred under our incubation conditions.

In control samples (no substrate present), there was a significant loss of totAsA after 24 h leading to a ~2-fold decrease in the size of the totAsA pool size in IG and red fruit discs of both cultivars (Additional file 3: Table S3). This presumably reflects losses due to AsA oxidation as a result of the stress associated with tissue wounding, and is similar to findings in blackcurrant [27] and apple [28]. Somewhat surprisingly however, the totAsA pool size of MG fruit discs remained essentially stable during the 24 h incubation period, suggesting an improved stress tolerance capacity for AsA recycling at this stage.

#### Non-radiolabelled substrate studies

**Figure 3:**
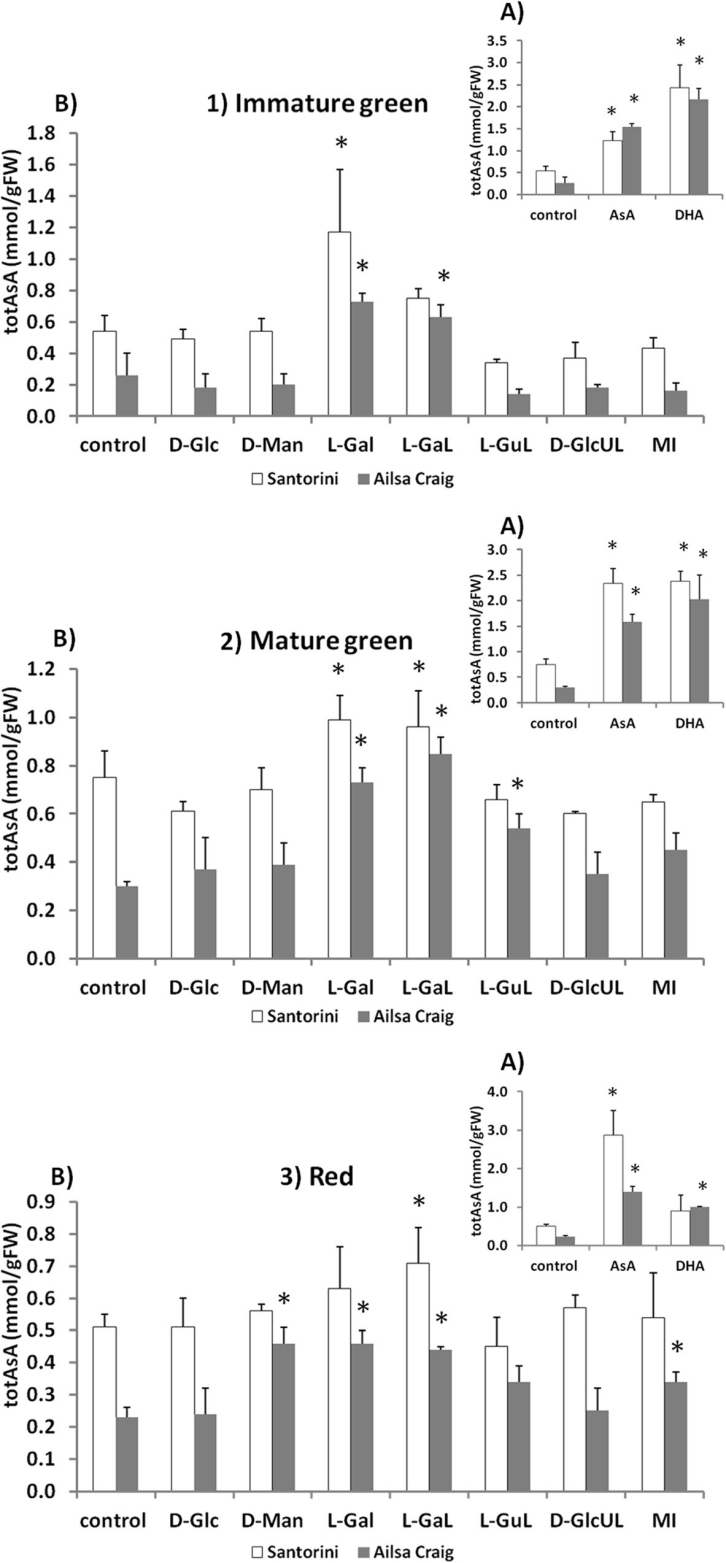
**Non-radiolabelled feeding experiments with L-ascorbic acid precursor substrates.** The effect of incubating with **A**) D-Glucose (D-Glc), D-Mannose (D-Man), L-galactose (L-Gal), and L-galactono-lactone (L-GaL) from the L-galactose pathway, L-gulono-lactone (L-GuL), D-glucurono-lactone (D-GlcUL), and *myo*-inositol (MI) from the alternative biosynthetic pathways, and with **B**) L-ascorbic acid (AsA) and dehydroascorbate (DHA), for 24 hours on totAsA concentrations (mmol/gFW) of 1) immature green, 2) mature green, and 3) red fruit discs of ‘Santorini’ (white) and ‘Ailsa Craig’ (gray). Results represent means of three replications ± SD, and asterisks indicate values that are significantly different from those of the control for 24 hours (*t*-test, P < 0.05).

Incubations of fruit discs with non-labelled AsA or DHA provides an indication of the uptake capacity of tissues for these different forms of vitamin C. The results show that in both cultivars, DHA is the preferred uptake form at the IG stage, and is also the preferred uptake form in MG fruit discs of ‘Ailsa Craig’ (Figure 3-1B and 2B). In red fruit discs however, AsA is most effective in increasing totAsA levels, resulting in an up to 6-fold increase relative to controls in both cultivars (Figure 3-3A; Additional file 3: Table S3). This suggests that the preference for reduced or oxidised AsA differs according to the fruit ripening stage. In addition, the rate of uptake of AsA and DHA was found to be noticeably higher in fruits of the low-AsA ‘Ailsa Craig’ cultivar early in development, indicating that the two cultivars have a different capacity to utilise external AsA sources (Figure 3A). This may reflect differences in the size and redox state of the AsA pool (‘Ailsa Craig’ fruits had generally lower totAsA-AsA concentrations and a higher % DHA than ‘Santorini’ fruits), differences in the capacity for endogenous AsA biosynthesis, or differences in the energetic requirements and capacity for transport of external AsA/DHA.

Pericarp tissues from both cultivars were capable of *de novo* AsA synthesis, but this capacity varied according to the substrate provided (Figure 3B; Additional file 3: Table S3). Incubations with 15 mM L-galactose (L-Gal) and/or L-galactono-1,4-lactone (L-GaL) lead to significantly enhanced tissue totAsA concentrations relative to control incubations at all ripening stages. In ‘Santorini’, the greatest increase was caused by incubations of IG fruit discs with L-GaL (2.2-fold), while in ‘Ailsa Craig’, by incubations of MG fruit discs with L-Gal (2.9-fold). In contrast, incubations with 15 mM D-glucose (D-Glc) or 15 mM D-mannose (D–Man) had no significant influence on totAsA levels with the exception of incubations of red fruit discs of ‘Ailsa Craig’ with D-Man. Precursors from the alternative AsA biosynthetic pathways [L-gulono-1, 4-lactone (L-GuL), or *myo*-inositol (MI)] also had only a limited effect on the totAsA pool size and only at specific ripening stages in ‘Ailsa Craig’ fruits. For instance, incubating MG and red fruit discs of ‘Ailsa Craig’ with L-GuL or MI, respectively, enhanced totAsA mean concentrations 1.8- or 1.5-fold, respectively, relative to the controls of 24 h (Figure 3B; Additional file 3: Table S3).

In general, these substrate incubation experiments increased the totAsA pool size more in the low-AsA ‘Ailsa Craig’ cultivar compared to the AsA-rich ‘Santorini’, and more in young fruits compared to red fruits in both cultivars. This may indicate that ‘Ailsa Craig’ is more substrate-limited than ‘Santorini’, or that the cultivars differ in their capacity for substrate uptake, similar to results observed for the uptake of AsA and DHA, possibly as a result of differences in fruit physiology/morphology. None of the incubations significantly influenced tissue totGSH-GSH concentrations at any developmental stage (data not shown).

#### Radiolabelled substrate studies; turnover and biosynthesis

**Figure 4:**
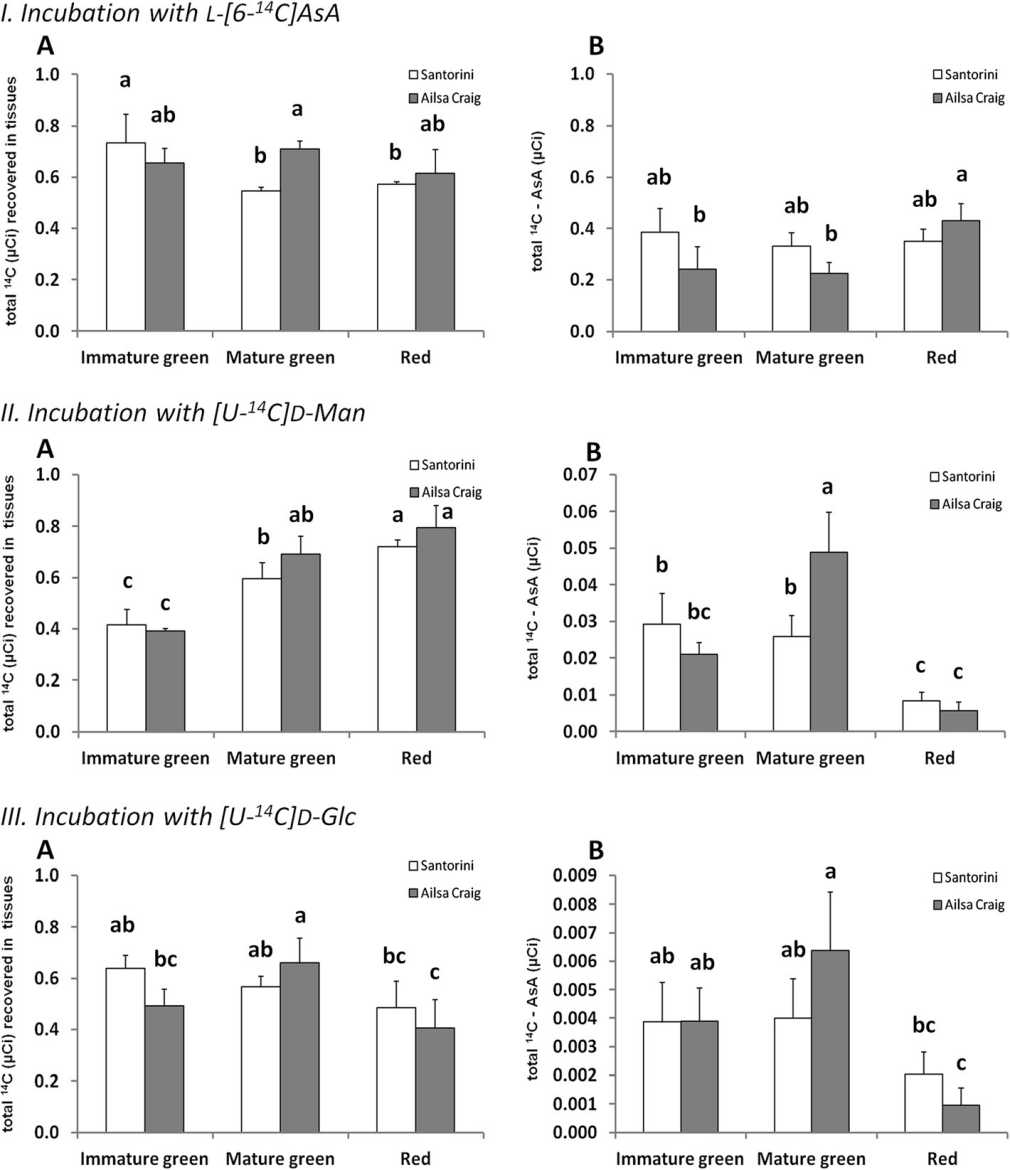
**Radiolabelled feeding experiments with L-ascorbic acid (AsA) precursor substrates.** Uptake and conversion of ^14^C in immature green, mature green and red fruit discs of ‘Santorini’ (white) and ‘Ailsa Craig’ (gray) after incubations for 24 hours with 1 μCi L-[6-^14^C]AsA (I), [U-^14^C]D-Mannose (U-^14^C]D-Man; II) and [U-^14^C]D-Glucose (U-^14^C]D-Glc; III). Results are represented as total radiolabel (μCi) recovered in tissues (**A**), and as total radiolabel (μCi) incorporated into total ^14^C-AsA (**B**). Data show the mean ± SD of three independent experiments. Different letters indicate significant differences (P < 0.05) between values based on Duncan's multiple-range-test in SAS.

Incubations of fruit discs with radiolabelled biosynthetic substrates allows us to determine their rate of uptake (total radiolabel recovered) and the rate of conversion to totAsA (radiolabel recovered as total ^14^C-AsA), without the possibility of substrate inhibition effects. Incubations with L-[6-^14^C]AsA was used to determine the net rate of uptake and turnover (breakdown) of the AsA pool as a significant proportion of total ^14^C-AsA radiolabel recovered was found in other metabolic products due to AsA degradation and/or conversion (Figure 4I-A and -B). Results show that there were no significant differences between the two cultivars in the uptake capacity of L-[6-^14^C]AsA over 24 h, with the exception of the MG stage (Figure 4I-A), where ~15% more total radiolabel was recovered from ‘Ailsa Craig’ pericarp discs than from ‘Santorini’ discs. However, from the amount of radiolabel recovered in total ^14^C-AsA, we see that the mean rate of turnover of the totAsA pool during ripening was higher in ‘Santorini’ than ‘Ailsa Craig’ at 13.8 and 10.9 nmol totAsA/gFW/h respectively (Table 1). In contrast, the % turnover was higher in ‘Ailsa Craig’ fruits prior to ripening at the IG and MG stages. In ‘Santorini’, the % turnover did not differ significantly across the different fruit developmental stages, and although the absolute turnover rates (nmol totAsA/gFW/h) were lower at the MG stage, the turnover rates at the red stage were as high as in young (IG) fruit. By comparison, in ‘Ailsa Craig’, turnover rates consistently decreased throughout development, and turnover rate was only 18% of the rate of ‘Santorini’ fruits at the red stage. This suggests that either AsA recycling is more efficient, or that in general the demand for AsA is lower in the ‘Ailsa Craig’ cultivar.

**Table 1:** Turnover rate (nmol/gFW/hour) and % turnover of total ascorbic acid (totAsA) pool in tomato fruits of ‘Santorini’ and ‘Ailsa Craig’ at different developmental stages

**Cultivar**	**Ripening stage**	**turnover rate of totAsA pool (nmol/gFW/hour)**	**% turnover of totAsA pool**
Santorini	Immature green	15.5 ± 3.21 a	39.23 ± 3.76 b
	Mature green	9.71 ± 1.23 b	39.50 ± 13.9 b
	red	16.2 ± 3.83 a	38.96 ± 9.86 b
Ailsa Craig	Immature green	6.86 ± 1.11 b	63.28 ± 4.02 a
	Mature green	8.56 ± 1.67 b	68.45 ± 6.11 a
	red	2.88 ± 0.93 c	30.07 ± 11.3 b

The % turnover of totAsA pool was calculated as the ratio of the mean % of total ^14^C-AsA recovered in the tissues and the mean % of total ^14^C taken up by the tissue following incubations with 1 μCi L-[6-^14^C]AsA for 24 h. Results are represented as mean values of three replications ± SD. Different letters indicate a significant difference (P < 0.05) between values at different ripening stages based on Duncan’s multiple-range-test in SAS.

Similar to results obtained with the L-[6-^14^C]AsA incubations, there were no significant differences between the cultivars in their rates of uptake of the other radiolabelled substrates at any developmental stage examined. The uptake of ^14^C-D-Man was found to increase around 2-fold with ripening (Figure 4II-A), while the uptake of ^14^C-D-Glc actually decreased slightly with ripening (Figure 4III-A). From the amount of total ^14^C radiolabel recovered as total ^14^C-AsA (i.e. rate of AsA biosynthesis), we can see that recoveries were dependent on both the cultivar and the developmental stage, as well as on the substrate itself (Figure 4II-B, and III-B). Also, as expected [1,27,28], [U-^14^C]D-Man was a more effective biosynthetic substrate than [U-^14^C]D-Glc. Comparing the two cultivars, there were no significant differences in the amount of [U-^14^C]D-Man radiolabel recovered as total ^14^C-AsA except at the MG stage, where incorporation was approximately twice as high in ‘Ailsa Craig’. Paradoxically, this is the stage where the greatest differences in totAsA (1.8-fold) - AsA (2.4-fold) concentrations exist between the two cultivars (Figure 2), suggesting that ‘Ailsa Craig’ could be substrate-limited at this stage. As reported in other systems [12,28,29], the rate of conversion of ^14^C-D-Glc was much lower than that of ^14^C-D-Man, with a maximum of only 0.6% recovered as total ^14^C-AsA in MG fruit in ‘Ailsa Craig’ (Figure 4III-B). However, the measured rates of AsA biosynthesis from ^14^C-D-Glc during development showed the same variations as observed for the incubations with ^14^C-D-Man, with highest rates for MG fruit from ‘Ailsa Craig’, and lowest rates at the red stage in both cultivars.

### Changes in the activity of AsA recycling and antioxidant enzymes during fruit ripening

Enzymes involved in AsA recycling and antioxidant defence were profiled throughout ripening. In all cases the activity profiles were expressed as units of activity per mg fresh weight (FW) since the profiles were identical to those obtained when activities expressed on the basis of soluble protein content (data not shown).

**Figure 5:**
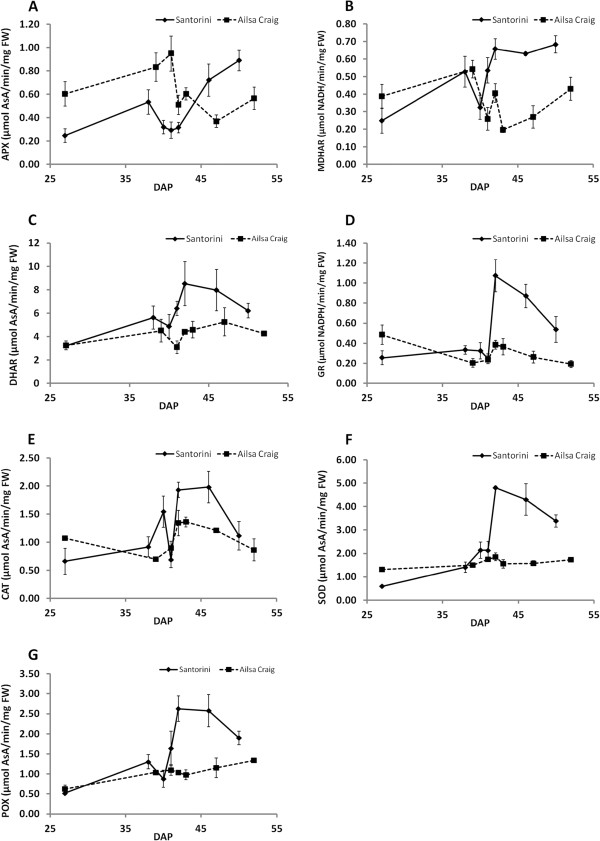
**Activities of antioxidant enzymes.** Changes in enzyme activities of ascorbate peroxidase (APX; **A**), monodehydroascorbate reductase (MDHAR; **B**), dehydroascorbate reductase (DHAR; **C**), glutathione reductase (GR; **D**), catalase (CAT; **E**), superoxide dismutase (SOD; **F**), and peroxidase (POX; **G**) based on protein content and fresh weight during ripening in ‘Santorini’ and ‘Ailsa Craig’ fruits grown in a hydroponic system in the greenhouse. Ripening stages are expressed as Days After Pollination (DAP). For ‘Santorini’: 27DAP, immature green (IG); 38DAP, mature green (MG); 40DAP, B-1; 41DAP, breaker (**B**); 42DAP, B + 1; 46DAP, pink; 50DAP, red. For ‘Ailsa Craig’: 27DAP, IG; 39DAP, MG; 41DAP, B-1; 42DAP, B; 43DAP, B + 1; 47DAP, pink; 52DAP, red. Results are represented as the mean of three replications ± SD. Each replication consisted of pooled samples as explained in materials and methods, and was the same used for AsA determination and gene expression analysis.

As with the results of substrate feeding experiments (Figure 3, 4), the two cultivars showed different activity profiles during ripening (Figure 5). Generally, the changes in activities throughout ripening were much smaller in ‘Ailsa Craig’, and prior to the breaker stage, all enzyme activities except ascorbate peroxidase (APX) were comparable in both cultivars. APX activity however was ~2-fold higher in ‘Ailsa Craig’ up to the B-1 stage. The main differences between the cultivars occurred after the breaker stage where all enzyme activities increased in ‘Santorini’, and remained substantially higher than in ‘Ailsa Craig’, suggesting an increased capacity to deal with oxidative stress in ‘Santorini’ fruits. This may help maintain the higher totAsA-AsA concentrations and lower % DHA (Figure 2) in red ripe fruits, and could represent part of a constitutive adaptive mechanism to the high-light, high-temperature and low-water conditions where ‘Santorini’ is normally cultivated.

The changes in AsA concentrations throughout the ripening process were partially reflected in the changes in enzyme activity measurements. For example, the activity of APX (P < 0.001), MDHAR (P < 0.001) and peroxidase (POX, P < 0.05) were correlated with AsA concentrations during ripening in ‘Santorini’ fruits, while the activity of APX (P < 0.05), catalase (CAT, P < 0.0001) and superoxide dismutase (SOD, P < 0.05) with AsA levels in ‘Ailsa Craig’ fruits (Additional file 4: Table S4), suggesting again that the two cultivars have different mechanisms to deal with oxidative stress. Of particular interest however were the changes in MDHAR activity around the breaker stage which followed the same characteristic pattern as changes in totAsA-AsA concentrations in ‘Ailsa Craig’ (Figure 2 and 5B). MDHAR is also the enzyme that displayed the most striking increase in activity at the breaker stage, increasing by 65% and 56% compared to B-1 in ‘Santorini’ and ‘Ailsa Craig’, respectively. This corresponds to an increase in AsA concentration by 17% and 73% in ‘Santorini’ and ‘Ailsa Craig’, respectively.

### Changes in the relative expression of AsA-related genes during fruit ripening

**Figure 6:**
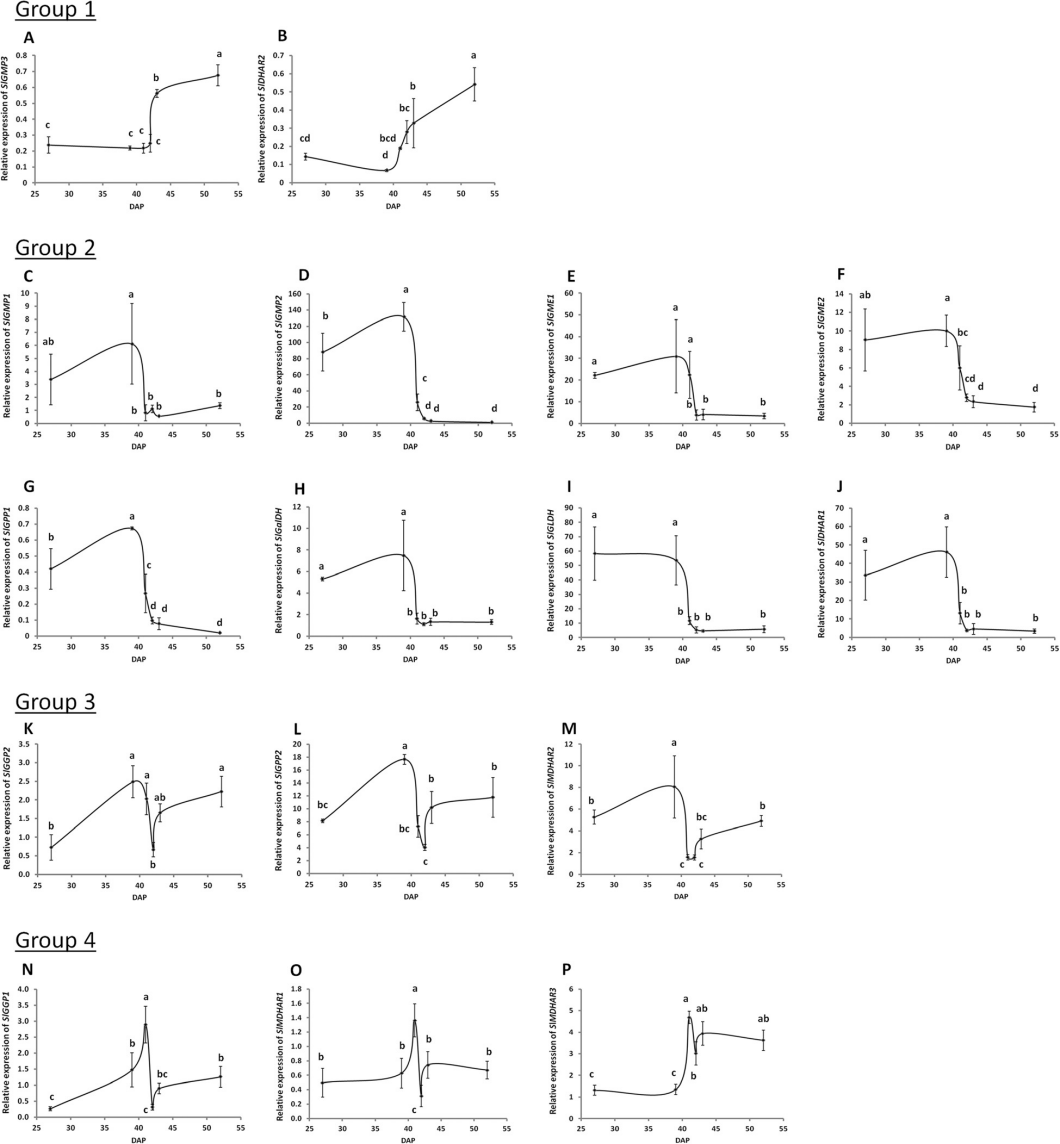
**Gene expression studies.** Changes in the relative expression of genes involved in L-ascorbic acid (AsA) metabolism of ‘Ailsa Craig’ fruits at immature green (IG; 27DAP), mature green (MG; 39DAP), B-1 (41DAP), breaker (B; 42DAP), B + 1 (43DAP), and red (52DAP) stage. Ripening stages are expressed as Days After Pollination (DAP). qPCR values were normalized against the geometrical mean of the expression of *CAC* and *PP2Acs*. Results are presented as the mean of three biological (each of them consist of a pool of 3 samples of equivalent stage) and two technical replications ± SD. Genes have been divided into four groups based on their relative expression pattern during ripening: 1) increase with ripening (**A**, *SlGMP3*; **B**, *SlDHAR2*), 2) decrease with ripening (**C**, *SlGMP1*; **D**, *SlGMP2*; **E**, *SlGME1*; **F**, *SlGME2*; **G**, *SlGPP1*; **H**, *SlGalDH*; **I**, *SlGLDH*; **J**, *SlDHAR1*), 3) peak at MG stage (**K**, *SlGGP2*; **L**, *SlGPP2*; **M**, *SlMDHAR2*) and 4) peak at B-1 stage (**N**, *SlGGP1*; **O**, *SlMDHAR1*; **P**, *SlMDHAR3*). Different letters indicate significant differences (P < 0.05) between values based on Duncan's multiple-range-test in SAS.

The expression of 16 tomato orthologues of genes from the L-galactose biosynthetic and the AsA-recycling pathways (Table 2) were studied in pericarp tissues of ‘Ailsa Craig’ fruits throughout ripening (Figure 6). Accurate real-time quantitative PCR (qPCR) results depend on the use of a suitable reference gene [30] whose expression does not significantly change during ripening. From the seven possible reference genes tested in our sample set, a clathrin adaptor complexes medium subunit (*CAC*) and a protein phosphatase 2A catalytic subunit (*PP2Acs*) showed the highest stability during ripening, with stability values of 0.344 and 0.369, respectively (Additional file 5: Table S5). Therefore all expression data were expressed relative to the geometric mean of these two genes, essentially as previously described [31].

**Table 2:** Tomato orthologues of candidate regulatory genes for L-ascorbic acid metabolism

**Gene**	**Reference Gene in*Arabidopsis***	**Unigene**	**Name**	**F-primer (5**^**′**^**-3**^**′**^**)**	**R-primer (5**^**′**^**-3**^**′**^**)**
**L-galactose pathway**					
GDP-mannose pyrophosphorylase	At2g39770	SGN-U568548	*SlGMP1*	CAGGTAGCATTATCGGTTGG	TTGATCTCCTTGTGGGGTAA
		SGN-U568547	*SlGMP2*	GTGTAGTTTTGCCCCACAAG	AGGAGAACTGGAAACCAACC
		SGN-U584300	*SlGMP3*	AGAAGCGACTGGGAGAGTTG	GAATCCGGTCCAAAACAGAA
GDP-mannose-3,5-epimerase	At5g28840	SGN-U581327	*SlGME1*	TTCCTGTCCAACACATTCCT	CTTTCTCGATCTGCTCCTTG
		SGN-U580362	*SlGME2*	ATTCGAGATGTGGGGAGATG	GGAAGGTTCTTGCCATCAAA
GDP-L-galactose phosphorylase	At4g26850	SGN-U579800	*SlGGP1*	TCCAAGTGTCGTTGCCATAA	TCGGAAGTAAGGGTTTGCTG
		SGN-U573852	*SlGGP2*	GCTCTCCATTTTGCCAGAGA	CCCCTTCCTTGCCAGTATTT
L-galactose-1-P-phosphatase	At3g02870	SGN-U600217	*SlGPP1*	TGGTGGCGCTGTAATAGTGA	AACGCTTCCTTGAGATGAGG
		SGN-U568299	*SlGPP2*	AGGTCCCTTCGTATGTGTGG	GAACCCTCCAGCTTCTTTCA
L-galactose dehydrogenase	At4g33670	SGN-U565558	*SlGalDH*	AGTAGCAACGACTGGAATGG	TGCACAACAGGAGATCACAA
L-galactonolactone dehydrogenase	At3g47930	SGN-U585649	*SlGLDH*	AGATTGAGGTTCCCAAGGAC	TTAGATAGGATGCGGTTTGG
**AsA recycling pathway**					
Monodehydroascorbate reductase	At1g63940	SGN-U583672	*SlMDHAR1*	CAAGGGTTTCGGTTCCTTCT	CTGCATTTCCTCCTCCAACT
	At3g52880	SGN-U573751	*SlMDHAR2*	AGATCGTTGGTGCATTCCTC	AAAACTGATGCCCTCCTGTG
	At3g27820	SGN-U584073	*SlMDHAR3*	AGGAATGGAATGTGCTGCTT	GACCGTGCCTTTCACAAACT
Dehydroascorbate reductase	At1g19570	SGN-U583361	*SlDHAR1*	AGGTGGCTCTTGGACACTTC	CTTCAGCCTTGGTTTTCTGG
	At5g16710	SGN-U577467	*SlDHAR2*	CACCCAGAGGGTTTTGCTTA	CTCCAGTGCCTGTGAGATGA

Details of the gene ID, the corresponding unigene, and the forward and reverse primers used in gene expression studies throughout ripening are shown.

The expression profiles during ripening allowed us to classify these orthologues into four groups (Figure 6) as follows; 1) genes whose expression increased dramatically during ripening, 2) genes whose expression decreased during ripening, 3) genes whose expression peaked at MG stage and 4) genes whose expression peaked one day before the breaker stage (Figure 6).

Profile group 1 contained orthologues of GDP-mannose pyrophosphorylase (*SlGMP3*, SGN-U584300) and of *DHAR* (*SlDHAR2*, SGN-U577467). Expression levels increased 3.4- (*SlGMP3*) or 6.1-fold (*SlDHAR2*) from MG to red stage in ‘Ailsa Craig’ fruits (Figure 6; group 1).

The majority of the AsA biosynthetic and recycling gene profiles were classified in group 2, where gene expression was high up until the MG stage, and then dropped sharply and remained low throughout ripening (Figure 6; group 2). This group includes orthologues of *GMP* (*SlGMP1*, SGN-U568548; *SlGMP2*, SGN-U568547), *GME* (*SlGME1*, SGN-U581327; *SlGME2*, SGN-U580362), *GPP* (*SlGPP1*, SGN-U600217), and *DHAR* (*SlDHAR1*, SGN-U583361), as well as L-galactose dehydrogenase (*SlGalDH*, SGN-U565558) and L-galactonolactone dehydrogenase (*SlGLDH*, SGN-U585649). In this profile group, expression levels decreased from 4.5- (*SlGMP1*) to 120-fold (*SlGMP2*) throughout ripening.

Genes of group 3 displayed short-term but dramatic changes in expression around the breaker stage (Figure 6; group 3). This group contains orthologues of *GGP* (*SlGGP2*, SGN-U573852), *GPP* (*SlGPP2*, SGN-U568299), and *MDHAR* (*SlMDHAR2*, SGN-U573751). Expression profiles were characterised by a peak at MG, followed by a decrease at the B-1 stage, and then a sharp increase at B + 1, followed by constant expression through the remainder of ripening.

The fourth group contains the other orthologue of the key biosynthetic enzyme *GGP* (*SlGGP1*, SGN-U579800), and two copies of the recycling gene *MDHAR* (*SlMDHAR1*, SGN-U583672; *SlMDHAR3*, SGN-U584073) (Figure 6). These transcripts proved to be the most interesting as their relative expression profiles showed the same characteristic changes around the breaker stage as observed in totAsA-AsA levels in ‘Ailsa Craig’, but with delay of one day (Figure 2).

To confirm the role of group 4 transcripts in explaining the cultivar-specific differences in totAsA-AsA concentrations, the expression of these three genes was also tested in red fruits of the AsA-rich cultivar (‘Santorini’). Here, qPCR values were normalized against CAC, as its expression was the most stable between red fruits of the two cultivars. Results clearly demonstrated that only the expression of *SlGGP1* was significantly higher (2-fold) relative to expression in red fruit of ‘Ailsa Craig’ (Table 3). However, while the relative expression of *SlMDHAR1* and *SlMDHAR3* did not differ significantly between cultivars, the mean MDHAR activities were 1.6-fold higher in ‘Santorini’ red fruits, which again suggests a role for AsA-recycling in maintaining elevated totAsA-AsA pools.

**Table 3:** Expression levels of key candidate genes in red fruit

					**Relative gene expression**				**Enzyme activity**	
**Cultivar**	**AsA (nmol gFW**^**-1**^**)**		***SlGGP1***		***SlMDHAR1***		***SlMDHAR3***		**MDHAR**	
Santorini	826.4 ± 32.3	a	2.448 ± 0.11	a	0.346 ± 0.13	a	1.222 ± 0.65	a	0.684 ± 0.05	a
Ailsa Craig	493.5 ± 7.29	b	1.233 ± 0.27	b	0.495 ± 0.26	a	1.268 ± 0.59	a	0.430 ± 0.07	b

Relative expression levels of *SlGGP1*, *SlMDHAR1* and *SlMDHAR3*, as well as MDHAR enzyme activity (μmol NADH min^-1^ mg FW^-1^) in ‘Santorini’ and ‘Ailsa Craig’ red fruits. qPCR values were normalized against *CAC*. Results are presented as the mean of at least four biological (each of them consist of a pool of three samples of equivalent stage) and two technical replications ± SD. Different letters indicate a significant difference (P < 0.05) between values based on Duncan's multiple-range-test in SAS.

## Discussion

### AsA and GSH concentrations in two tomato cultivars

Fully ripe tomato fruits have only ‘moderate’ totAsA-AsA concentrations [1] compared to the fruits of other species such as blackcurrant [27] and kiwifruit [9]. Under the standardised hydroponic greenhouse conditions used here, fruit AsA concentrations of ‘Santorini’ and ‘Ailsa Craig’ at the red ripe stage were 14.6 and 8.69 mg/100gFW, respectively, representing a 1.7-fold difference between the cultivars. This is identical to the differences observed between the same cultivars when grown under field conditions in Greece [25], indicating that there is a strong and stable genetic basis for the regulation of the AsA pool size in tomato. However, the field-grown tomatoes had about 1.5-fold higher fruit AsA levels compared to greenhouse-grown tomatoes, presumably due to the higher levels of irradiance (and temperature) experienced in the field [32]. The differences in totAsA concentrations of red ripe fruits however were not as great (21.2 and 17.7 nmol/gFW in ‘Santorini’ and ‘Ailsa Craig’, respectively) due to the fact that the AsA pool in ‘Ailsa Craig’ contained a higher % DHA (i.e. it was more oxidised). This is possibly due to the lower activities of the enzymes involved in AsA recycling at this stage (Figure 5).

On average, totAsA, AsA, and GSH concentrations were 1.8-, 2.4-, and 1.4-fold higher in ‘Santorini’ fruit than ‘Ailsa Craig’ throughout ripening. However, the concentrations of totGSH-GSH did not vary significantly during ripening. Since the time to ripening was similar in both cultivars and there were also no significant differences in fruit water contents it is unlikely that the changes are related to a metabolite ‘dilution’ effect (Additional file 1: Table S1). These differences therefore represent clear genetic differences in the capacity to accumulate fruit totAsA-AsA.

### AsA and GSH accumulation in tomato fruits during ripening

The accumulation of totAsA during fruit ripening has been well studied in several plant species, and reports show that patterns vary according to the species and the cultivar studied, as well as growth conditions. For example in fruits of different tomato cultivars, totAsA concentrations were shown to both increase [11,18,33,34], or to remain essentially unchanged [35] during ripening. The two cultivars used here also showed different patterns of fruit totAsA-AsA accumulation even under identical growth conditions. Nevertheless, one feature common to both cultivars was a characteristic spike in totAsA-AsA concentrations at the breaker stage, which proved useful to help understand the factors involved in regulating AsA concentrations during ripening. The breaker stage in tomatoes (when fruit colour first begins to change from green to red) marks the onset of ripening linked to the induction of ethylene–related metabolic pathways, and leads to a major shift in fruit metabolism. The biological basis for this spike in AsA (and GSH) concentrations is unclear, but an increase in H_2_O_2_ accumulation at the breaker stage has previously been reported in tomatoes [33]. While H_2_O_2_ levels were not measured here, the enhancement of the size of the totAsA-AsA pool could be indicative of an adaptive response to such an increase in H_2_O_2_ concentrations.

In ‘Ailsa Craig’, the breaker stage was associated with a 15% decrease in % DHA and a 48% decrease in % GSSG, due to an increase in both AsA and GSH concentrations. Taken together with the 56% increase in fruit MDHAR activity in ‘Ailsa Craig’ fruits relative to the B-1 stage, these results suggests that the enhanced capacity for AsA recycling could be linked to the observed increases in AsA and GSH around these stages. Unfortunately we do not have data on changes in the AsA biosynthetic capacity (substrate incubations) at this specific time point as there were insufficient fruit to carry out all incubations. However, similar results were observed in ‘Santorini’, where a 65% increase in MDHAR activity was correlated with an increase in the AsA pool at the breaker stage. In fact, MDHAR was the only enzyme whose activity was associated with the increase in fruit totAsA-AsA levels of both cultivars at the breaker stage, and although activity results represent the mean activities of all subcellular isoforms, the results are supported by changes in the expression of *SlMDHARs* in ‘Ailsa Craig’ *–* see discussion here under.

Previous work on GSH levels in tomato fruits has shown that concentrations increase during the later stages of ripening, but that changes in GSH are not correlated with changes in AsA [33]. Our results support these conclusions, and we see that both totGSH and GSH concentration profiles differ from the totAsA-AsA profiles during ripening in both cultivars, suggesting that these antioxidant pools are differently regulated.

### AsA uptake, biosynthesis and recycling in tomato fruits

Earlier studies in foliar cell suspension cultures indicate that the preferred uptake form of AsA is as the oxidized form, DHA [13,36]. Our results with young tomato fruits of both cultivars support this, but by the end of ripening at the red stage there was a clear preference for AsA, which increased the size of the totAsA pool 3.3-fold more than incubation with DHA in ‘Santorini’, and 1.4-fold more in ‘Ailsa Craig’. These different responses of the cultivars may be related to the less oxidized redox status of the totAsA pool in ‘Santorini’ ripe fruits, or differences in the energetic requirements and capacity for transport of external AsA/DHA as reported in bean seedlings [37].

The ability of fruit tissues to synthesize AsA *de novo* has been demonstrated by substrate feeding experiments in several fruit species, including courgette [38], apple [28,39], and blackcurrant [27]. Here, the highest accumulation of intercellular totAsA-AsA in both tomato cultivars took place following incubations of fruit discs with L-Gal or L-GaL. This is to be expected as these are the substrates for the last two enzymatic steps of the L-galactose pathway (Figure 1). The results also show that when supplied with an external carbon source, substrates of the L-Gal pathway are more effective than precursors of alternative biosynthetic routes at increasing intercellular totAsA-AsA concentrations. Nonetheless, incubations with L-GuL or MI were also able to increase totAsA levels of MG and red fruits, respectively, but only in the low-AsA cultivar ‘Ailsa Craig’. Incubations with L-GuL have previously been reported to effectively stimulate AsA levels in *Arabidopsis* leaf suspension cultures [13] and in apple fruits [28], while overexpressing *myo*-inositol oxygenase in *Arabidopsis*, the gene encoding the enzyme responsible for the conversion of MI to D-glucuronate (Figure 1), supports a role for MI as a potential precursor of AsA, at least in foliar tissues [16]. In agreement with previous reports in apple [40], incubations with D-glucuronolactone (D-GlcUL) did not increase fruit totAsA levels at any ripening stage in either cultivar, however little is known about the efficiency of uptake or feedback inhibition effects of this substrate. Therefore, the existence or functionality of alternative AsA biosynthetic pathways seems to be cultivar and/or developmental stage-specific.

Young fruits (IG, MG) of both cultivars displayed higher AsA biosynthetic capacities than mature fruits, presumably to help support higher rates of cellular metabolism during the stages of fruit cell division and expansion. Despite this, red ripe fruits had higher AsA concentrations (Figure 2B). Similarly, despite having lower totAsA-AsA concentrations, ‘Ailsa Craig’ fruits had a higher AsA biosynthetic capacity than ‘Santorini’. Therefore biosynthetic capacity is not related to totAsA-AsA concentrations. The lower biosynthetic capacities of the high versus the low-AsA cultivar may represent the results of a feedback inhibition of AsA on AsA biosynthesis in ‘Santorini’ fruits, or could be due to a lower demand for AsA in these ‘Santorini’ fruits, or differences in uptake capacities. Feedback regulation of AsA pool has been previously observed in spinach [41], as well as in pea seedlings [29], where the activity of GLDH, the last enzyme in the L-galactose biosynthetic pathway, was competitively inhibited by increased AsA levels.

Since differences in the rate of AsA biosynthesis do not correlate with cultivar-specific differences in fruit totAsA-AsA concentrations, or the changes observed during ripening, the accumulation of totAsA-AsA is possibly due to differences in the rate of AsA turnover and AsA recycling. Indeed, our results indicate that both of these mechanisms significantly influence the size of the fruit totAsA pool. For example, the % turnover of the AsA pool was significantly higher (2-fold) in young (low AsA) compared to the red (high AsA) fruits in ‘Ailsa Craig’, and 1.7-fold lower in ‘Santorini’ young fruits compared to ‘Ailsa Craig’ fruits at the same developmental stage. Nevertheless, the actual turnover rate was higher in ‘Santorini’ fruits, again indicating a possible link between the size and the rate of turnover of the AsA pool [29]. On the other hand, totAsA pool is smaller and more oxidized in ‘Ailsa Craig’ (Figure 2C) which is also the cultivar with a lower capacity for AsA recycling (Figure 5). Interestingly, of all the enzymes measured, it is the enhanced MDHAR activity that is associated with the increased totAsA–AsA concentrations measured at the breaker stage in both cultivars.

To summarize this section therefore, AsA biosynthetic capacities are higher early in fruit development, even though concentrations of AsA (in both cultivars) and totAsA (only in ‘Ailsa Craig’) are higher in the later stages of ripening. The lack of accumulation of totAsA-AsA at IG fruits is due to higher rates of turnover of the AsA pool. The two cultivars also differ in net *de novo* biosynthetic capacities, with the ‘low-AsA’ ‘Ailsa Craig’ actually displaying a higher biosynthetic capacity. Again, this is compensated for by a higher % turnover of the AsA pool early in fruit development, and a lower rate of AsA recycling, as manifested by a more oxidized redox status compared to ‘Santorini’.

### Candidate genes for AsA regulation throughout ripening

Similar to results in kiwi [9] and tomato [11], we found high relative expression of most of the biosynthetic genes from the L-galactose pathway early in fruit development (Figure 6, group 2 and 3). This correlates with the higher biosynthetic capacities measured and may reflect increased requirements of AsA for cell division/expansion [42]. In a previous study, the enhanced expression of *GPP*, as monitored by northern blot analysis, has been correlated with increased AsA concentrations during the later stages of the ripening process in ‘Ailsa Craig’ fruits [11]. However, no such correlation between *GPP* expression and AsA levels was observed by us during ‘Ailsa Craig’ fruit ripening. In fact of all genes examined, only the relative expression patterns of the ‘group 4’ genes (*SlGGP1*, *SlMDHAR1* and *SlMDHAR3*) (Figure 6) were strongly correlated (P < 0.001) with the changes in fruit totAsA-AsA levels around the breaker stage, with a delay of one day (Additional file 6: Table S6). A similar delay between gene expression and changes in AsA concentrations was noticed in tomato leaves [26]. Therefore these three genes seem to represent good candidates for the regulation of fruit AsA concentrations. Significant correlations were also found between the expression of *SlGMP2* and *SlGGP2* and AsA concentrations around this stage (P < 0.05), but not with totAsA concentrations (Additional file 6: Table S6), indicating that these transcripts may also play a role in regulating the size of the reduced AsA pool.

GGP (VTC2) represents the first committed step of AsA biosynthesis, and has previously been suggested to be a key rate-limiting step in plants [5,9,10,43]. Recent results from our lab indicate that paralogues of apple *GGP* are key regulators of totAsA-AsA concentrations in fruits [10], and others have shown that ectopic expression of the single kiwifruit *GGP* orthologue results in enhanced totAsA contents in both transgenic tomato (3- to 6-fold increase) and strawberry (2-fold increase) [5]. Therefore, our results here support a central role for *GGP* in the regulation of fruit AsA pool in fruits of several different plant species. In addition, although we cannot exclude the possibility that other genes may play a role in regulating fruit AsA pool size in the high AsA cultivar during ripening, differences in *SlGGP1* expression could also help to explain the differences in fruit totAsA-AsA concentrations between cultivars, as expression levels were twice as high in red ripe fruits of *‘*Santorini’ compared to the low-AsA cultivar, ‘Ailsa Craig’ (Table 3). These results should however be further validated by testing *SlGGP1* expression in fruits of a larger collection of tomato cultivars with a wider range of totAsA-AsA concentrations.

On the basis of Quantitative Trait Loci (QTL) studies in several tomato populations, Stevens and co-authors have proposed a role for an orthologue of *MDHAR*, as a candidate gene for the genetic control of AsA in tomato fruits [22,23]. This candidate gene corresponds to *SlMDHAR2* (SGN-U573751), but gene expression profiles here do not support this conclusion, and others have shown that overexpression of *SlMDHAR2* in tomato can even lead to a moderate decrease in AsA levels of MG fruits [24]. However, MDHAR activity is well correlated with AsA concentration in ‘Santorini’ fruits during ripening (Additional file 4: Table S4), and is linked to the short-term changes in totAsA-AsA around the breaker stage in ‘Ailsa Craig’ fruits (Figure 5), while expression of the other two *MDHAR* orthologues, *SlMDHAR1* (SGN-U583672) and *SlMDHAR3* (SGN-U584073), do closely mirror changes in totAsA-AsA concentrations in ‘Ailsa Craig’ fruits around this stage (Figure 6). In contrast, expression levels of the two orthologues of the other main AsA-recycling gene *DHAR*, did not reflect changes in totAsA-AsA changes (Figure 6).

Although the results presented here indicate a role for the *SlMDHAR1* and *SlMDHAR3* gene products in the control of AsA pool during ripening in ‘Ailsa Craig’, their relative expression levels did not differ significantly between the two cultivars at the red stage. Despite this, MDHAR enzyme activity was actually 1.6-fold higher in fruits of the high-AsA cultivar. This discrepancy between gene expression and enzyme activity is possibly related to the fact that activity results represent the mean activities of all subcellular isoforms, while expression results represent the expression of individual members of the gene family.

## Conclusions

In this work, we set out to try and unravel the regulation of AsA metabolism during fruit ripening in two tomato cultivars with different fruit totAsA-AsA levels, and so to identify key regulatory elements. Substrate feeding experiments show that as in other fruit species, young tomato fruits have a higher capacity to accumulate and synthesize AsA before the onset of ripening (IG and MG fruits), and that biosynthesis occurs primarily via the L-galactose biosynthetic pathway. However, at these early developmental stages, despite an enhanced biosynthetic capacity, fruits do not accumulate high totAsA-AsA concentrations due to a higher rate of breakdown of the AsA pool (‘Ailsa Craig’) perhaps as a result of the enhanced need for AsA in cell expansion of the developing tissue, or due to low AsA recycling activities (‘Santorini’).

In both cultivars (but to a greater extent in ‘Ailsa Craig’), totAsA and AsA concentrations increase significantly later on in ripening and our results suggest that this is due to increased recycling and/or decreased breakdown again depending on the cultivar. Comparing the two cultivars, increases in AsA recycling capacities after the breaker stage seem to be responsible for the enhanced totAsA-AsA levels in fruits of the high-AsA cultivar (‘Santorini’), even though it possesses lower net biosynthetic capacity than the low-AsA cultivar (‘Ailsa Craig’) at the same developmental stage. This lower biosynthetic capacity could be due to either lower uptake capacities for AsA biosynthetic precursors, or due to feedback inhibition of AsA biosynthesis by increased levels of AsA itself. One previously unrecognized characteristic of both cultivars is a sharp peak in fruit totAsA-AsA concentrations at the breaker stage. This peak could be of commercial relevance since tomatoes are typically picked at the breaker stage and then allowed to ripen off-vine.

From the range of AsA metabolic genes examined, only the levels of *SlGGP1* and of the two gene copies of *SlMDHAR* correlated with changes in totAsA-AsA concentrations during ripening in ‘Ailsa Craig’. At the red stage however only differences in the expression of *SlGGP1* were sufficient to explain totAsA-AsA levels, although MDHAR enzyme activity was higher in ‘Santorini’ fruits. Therefore it seems that fruit totAsA-AsA concentrations are regulated by changes in both biosynthetic and recycling activities, and that the relative contribution of these mechanisms depends on both the cultivar and developmental stage. Identification of the functional alleles of these candidates will enable us understand their relative contribution to fruit AsA regulation and can lead to the development of molecular markers suitable for breeding and selection.

## Methods

### Plant material and growth

Tomato plants (*Solanum lycopersicum*; cv. ‘Ailsa Craig’ and cv. ‘Santorini’) were cultivated using standard greenhouses practices in a hydroponic system in summer 2008 at the University’s greenhouses in KU Leuven, Belgium. Plants were grown at 20°C under 16 h supplemental lighting (when sunlight is less than 250 W/m^2^, supplemental lighting of 75 W/m^2^ was applied), followed by 8 h dark conditions at 18°C. The plants received a complete nutrient solution (Additional file 7: Table S7) for which electrical conductivity (EC) was maintained at 2.0 ± 0.2 dS · m^–1^ and pH at 6.0 ± 0.3, while the frequency of irrigations was adjusted according to the EC and pH of the leached solution. Only 5 trusses per plant and a maximum of 3 fruits per truss were kept in order to accelerate ripening and ensure homogenous fruit quality.

### Sampling

All fruits were harvested between 28^th^ of August and 15^th^ of September 2008. A minimum of ten fruits of the same ripening stage for each of the two cultivars were sampled randomly from different trusses of 20 individual plants over this period. Fruits were initially tagged at 7 days after pollination (DAP) and harvested at one of the following stages: immature green (IG), mature green (MG), one day prior breaker (B-1), breaker, one day after breaker (B + 1), pink and red stage. For ‘Santorini’ fruits, the above stages corresponded to 27, 38, 40, 41, 42, 46 and 50 DAP, respectively, and for ‘Ailsa Craig’ fruits to 27, 39, 41, 42, 43, 47 and 52 DAP, respectively, under our greenhouse conditions. The first appearance of orange colour (carotenoid biosynthesis) on the external surface of the fruit was used to define the breaker stage. To ensure developmental synchrony, fruits were inspected both internally (seed and locular jelly development, pigmentation) and externally (size, shape, skin colour), and only those appearing of equivalent stage were kept for further analysis. At each developmental time point, pericarp tissues of the harvested fruits collected over a period of 18 days were combined in three pools with a minimum of three fruits each, and immediately frozen in liquid nitrogen. Each developmental pool contained fruit harvested at different dates (but at the same physiological stage), eliminating variations due to differences in environment over the experimental period.

### AsA and GSH measurements

Reduced AsA and GSH were measured by HPLC essentially as described by Davey et al. [44]. Extracts were filtered and injected into an HPLC system (Waters 2690 separation module) equipped with a C_18_ rocket column (GRACE, 53 mm x 7 mm). AsA and GSH were detected at 243 nm or 197 nm, respectively (Waters 996 photodiode array detector).

### Determination of AsA biosynthetic capacity

The capacity of AsA biosynthesis was investigated by incubating 1-cm-diameter fruit pericarp fruit discs with non-radiolabelled sugar precursors of AsA biosynthesis, and analysing the subsequent changes in antioxidant contents as a function of incubation time by HPLC [12,44]. Incubations were carried out using IG, MG and red fruits from cv. ‘Santorini’ and cv. ‘Ailsa Craig’. The following precursor molecules were used: D-Glc, D-Man, L-Gal, L-GaL, L-GuL, D-GlcUL and MI, as well as AsA and DHA, all at a concentration of 15 mM (Sigma-Aldrich). Incubations were carried out at a pH of 6.8, using 20 mM MES buffer and 200 mM D-mannitol as osmoticum. All incubations were performed at 25°C, under continuous light for 24 h. Control samples for 24 h consisted of the same incubation medium without the addition of sugar precursors of AsA biosynthesis. Three replications were used for each sample set.

Radio-labelled studies were carried out using 1 cm diameter fruit pericarp fruit discs (same stages as before) incubated with 1 *μ*Ci of L-[6-^14^C]AsA, [U-^14^C]D-Man and [U-14C]D-Glc, all with a specific activity of 50 *μ*Ci/250 *μ*L (Amersham Biosciences Europe GmbH, Rosendaal, The Nederlands). All incubations were carried out at room temperature (~25°C) under continuous light for 24 h. Discs were then washed three times with sterile iso-osmotic solution and AsA extracted for HPLC analysis as above. Radiolabel in the extracts was detected online using a Ramona 2000, Radioactivity detector (Raytest Isotopenmeßgeräte, GmbH), fitted with an 80-μL solid scintillator detection cell connected serially after the UV-detector [44].

### Enzyme extractions and assays

Frozen fruit tissue samples of the pooled biological replicates were crushed to a fine powder in liquid nitrogen. Total protein were extracted from 3–4 g of homogenised tissues with the same volume of a phosphate buffer (50 mM, pH 7.6) containing 1 mM EDTA, 0.3% Triton X-100, 1% polyvinylpolypyrrolidone (PVPP) and 10% glycerol, as well as freshly prepared 0.5 mM AsA and 1 mM dithiothreitol (DTT) in the case of APX assay. The homogenates were then centrifuged at 14,000 x g for 20 min at 4°C, and the supernatants were passed through an ion-exclusion Sephadex G-25 column (PD 10, GE Healthcare), equilibrated with 25 ml 0.1 M phosphate buffer (pH 7.4), containing 1 mM EDTA and 10% glycerol, before being stored in aliquots at −80°C. The protein content of the extracts was quantified in triplicate as according to Bradford’s method [45].

The activity of APX (EC 1.11.1.11) was measured by monitoring the decrease in absorbance at A_290_, as adapted from Nakano and Asada [46]. The activity of MDHAR (EC 1.6.5.4) was assayed by measuring the decrease in absorbance at 340 nm [47], while the activity of DHAR (EC 1.8.5.1) was assayed by monitoring the GSH-dependent production of AsA, and the rate of non-enzymatic DHA reduction was subtracted [47]. The activity of glutathione reductase (GR; EC 1.6.4.2) was assayed by following the decrease in absorbance at 340 nm due to GSH-dependent oxidation of NADPH [48]. Catalase (CAT; EC 1.11.1.6) activity was assayed by directly measuring the rate of H_2_O_2_ degradation at 240 nm every 25 s for 2 min. Superoxide dismutase (SOD; EC 1.15.1.1) activity was measured in a reaction solution containing 50 mM Pipes buffer, pH 7.5, 0.4 mM o-dianisidine, 0.5 mM diethylenetriamine pentaacetic acid, and 26 μM riboflavin, following the absorbance at 450 nm for 15 min. Pyrogallol peroxidise (POX) activity was measured based on a method described by Chance and Maehly [49].

### Relative gene expression of AsA-related candidate genes in tomatoes

Structural genes involved in plant AsA metabolic pathways (biosynthesis and recycling) were selected as potential regulatory genes for fruit AsA concentrations in tomatoes. The sequences of genes from *Arabidopsis* or fruit species were retrieved from GenBank ( http://www.ncbi.nlm.nih.gov/genbank/), or PLAZA 2.0 ( http://bioinformatics.psb.ugent.be/plaza/), and orthologous sequences were searched for in the tomato reference genome ( http://solgenomics.net). The aminoacid sequences of all orthologues were then aligned in MUSCLE using the BLOSUM62 matrix and phylogenetic trees constructed using neighbour-joining method in GENEIOUS software version 5.6.3 [50] in order to identify tomato orthologs for each *Arabidopsis* accession.

Gene expression studies were performed following MIQE (Minimum Information for publication of Quantitative Real-Time PCR Experiments) guidelines [30]. Total RNA was isolated from flesh fruit tissues using a modified CTAB method [51]. Purity of total RNA extracted was determined as the 260/280 nm and 260/230 nm ratio with NanoDrop 2000 (Thermo Scientific), and integrity was checked by electrophoresis in 1% agarose gel (Gel Doc EZ Imager by Bio-Rad). One μg of total RNA was reverse transcribed to cDNA with Superscript II (Invitrogen) using random oligo d(T) primers. Gene-specific primer pairs (Table 2) were designed using Primer3 software version 4.0 ( http://frodo.wi.mit.edu/primer3/) employing the following conditions: product size between 75 to 200 bp with optimum 120 bp; GC% ranging from 40-55%; self complementarity and 3’ self complementarity were set at 4 and 1, respectively; and with all the other parameters set at default.

The expression profiles of AsA-related genes during fruit ripening were determined by real-time quantitative PCR (qPCR) using SYBR Green I technology on a Rotor Gene Q (Qiagen). The stability of expression of seven published reference genes was evaluated in our sample set (Additional file 5: Table S5) using NormFinder software ( http://www.mdl.dk/publicationsnormfinder.htm). All reactions were set up in duplicate containing: 1 μl cDNA template (50 ng), 7.5 μl Absolute^TM^ QPCR SYBR Green Mix and 1 μl of each primer (3.75 mM) in a final volume of 15 μl. The cycling conditions were as follows: denaturation step at 95°C for 10 min, followed by 45 cycles of denaturation at 95°C for 20 s, annealing at 63°C for 20 s and extension at 72°C for 20 s. A melting curve analysis, ranging from 55°C to 95°C, with temperature increasing steps 0.5°C/s was used to confirm specificity of amplifications. Criteria of acceptance for reaction efficiency ranged from 0.83 to 1.03 and both R and R^2^ > 0.97. For each run, a standard curve based on cDNA pooled from all samples to be analyzed in a range of different dilutions was included. The relative quantification of expression levels was performed using the comparative C_T_ method [52]. Two technical replications were performed for each of the three biological replications per sample. All expression data was calculated as an expression ratio relative to the geometric mean of the two most stable reference genes [31].

### Descriptive statistical analysis

Statistical analysis of all traits was carried out using the SAS 9.2 software package (SAS Institute). Duncan’s multiple test at 5% was used to evaluate differences between cultivars, and/or different stages, and Pearson correlation coefficients were employed to compare correlations between antioxidant traits, enzyme activities and gene expression during ripening.

## Abbreviations

*APX*: ascorbate peroxidise; *AsA*: L-ascorbic acid; *B*: breaker; *CAC*: clathrin adaptor complexes medium subunit; *CAT*: catalase; *DAP*: days after pollination; *DHA*: dehydroascorbate; *DHAR*: dehydroascorbate reductase; *D-Glc*: D-glucose; *D-GlcUL*: D-glucuronolactone; *D-Man*: D-mannose; *L-Gal*: L-galactose; *L-GaL*: L-galactono-1,4-lactone; *L-GuL*: L-gulono-1,4-lactone; *IG*: immature green; *GalDH*: L-galactose dehydrogenase; *GGP*: GDP-L-galactose phosphorylase; *GLDH*: L-galactonolactone dehydrogenase; *GME*: GDP-mannose 3,5-epimerase; *GMP*: GDP-mannose pyrophosphorylase; *GPP*: L-galactose-1-phosphate phosphatise; *GR*: glutathione reductase; *GSH*: glutathione; *GSSG*: oxidized glutathione; *MDHA*: monodehydroascorbate; *MDHAR*: monodehydroascorbate reductase; *MG*: mature green; *MI*: *myo*-inositol; *POX*: peroxidise; *PP2Acs*: protein phosphatase 2A catalytic subunit; *SOD*: superoxide dismutase; *totAsA*: total AsA; *totGSH*: total GSH.

## Competing interests

The authors declare that they have no competing interests.

## Authors’ contributions

IM designed the experiment, grew tomato plants and sampled fruits, quantified metabolites, carried out substrate feeding experiments, measured enzyme activities, performed gene expression studies and wrote the manuscript. JK supervised the experiments and edited the manuscript. AKK helped design the experiments, and edited the manuscript. MWD designed the experiments, helped with the interpretation of the results and largely contributed to the manuscript revision. All authors read and approved the final manuscript.

## Supplementary Material

Additional file 1 ** Table S1.** Fresh weight (g), % water content, and total soluble solids (°Brix) in entire tomato fruits of ‘Santorini’ and ‘Ailsa Craig’ at different ripening stages. Ripening stages: IG, Immature Green; MG, Mature Green; B-1, Breaker −1 day; B, Breaker; B + 1, Breaker + 1 day; PK, Pink; R, Red. Results are represented as mean measurements in 10 tomato fruits ± SD. Click here for file

Additional file 2 ** Table S2.** Determination of L-ascorbic acid and glutathione during fruit development and ripening. Changes in total ascorbic acid (totAsA), L-ascorbic acid (AsA), total glutathione (totGSH), and glutathione (GSH) concentrations (nmol/gFW) during fruit development and ripening of ‘Santorini’ and ‘Ailsa Craig’ fruits. Ripening stages: IG, Immature Green; MG, Mature Green; B-1, Breaker −1 day; B, Breaker; B + 1, Breaker + 1 day; PK, Pink; R, Red. Results are represented as the mean of three biological replications. Statistical analysis was carried out for each cultivar independently. Means of each cultivar with the same letter are not significantly different (a = 0.05) based on Duncan’s multiple-range-test in SAS. Click here for file

Additional file 3 **Table S3. **Non-radiolabelled feeding experiments with AsA precursor substrates. The effect of incubating with D-glucose (D-Glc), D-mannose (D-Man), L-galactose (L-Gal), and L-galactono-lactone (L-GaL) from the L-galactose pathway, L-gulono-lactone (L-GuL), D-glucurono-lactone (D-GlcUL), and *myo*-inositol (MI) from the alternative biosynthetic pathways, or AsA itself and DHA for 24 hours on total AsA (totAsA) concentrations (mmol/gFW) of immature green (IG), mature green (MG), and red fruit discs of ‘Santorini’ and ‘Ailsa Craig’. Results represent means of three replications ± SD, and asterisks indicate values that are significantly different from those of the control for 24 hours (*t*-test, P < 0.05). Click here for file

Additional file 4 ** Table S4.** Correlations between ascorbic acid and enzyme activities of ‘Santorini’ and ‘Ailsa Craig’ fruits during ripening. Pearson correlation coefficients between ascorbic acid (AsA), total ascorbic acid (totAsA), % dehydroascorbate (% DHA) and enzyme activities of ascorbate peroxidise (APX), monodehydroascorbate reductase (MDHAR), dehydroascorbate reductase (DHAR), glutathione reductase (GR), catalase (CAT), superoxide dismutase (SOD), and peroxidase (POX) in ‘Santorini’ and ‘Ailsa Craig’ fruits during ripening. *P < 0.05, **P < 0.01, ***P < 0.001, ****P < 0.0001, n.s. not significant. Click here for file

Additional file 5 ** Table S5.** List of primer sequences of the reference genes tested for stability across ripening stages of ‘Ailsa Craig’ fruits. The stability values for the reference genes were evaluated in ‘Ailsa Craig’ fruits throughout ripening using the NormFinder software. Click here for file

Additional file 6 ** Table S6. **Correlations between ascorbic acid and gene expression of ‘Ailsa Craig’ fruits around the breaker stage. Pearson correlation coefficients of the expression of AsA-related genes from biosynthetic and recycling pathways (listed in Table 2) measured at one day before breaker, breaker and one day after breaker stages, with ascorbic acid (AsA), and total ascorbic acid (totAsA) measured at the breaker, one day after breaker and two days after breaker stages i.e. assuming a delay of one day between gene expression and changes in fruit AsA concentrations. *P < 0.05, **P < 0.01, ***P < 0.001, ****P < 0.0001, n.s. not significant. Click here for file

Additional file 7 ** Table S7. **Composition of the nutrient solution used in the hydroponically-grown tomatoes. Click here for file
