# Supplementary material for: Regulation of fruit ascorbic acid concentrations during ripening in high and low vitamin C tomato cultivars
Source: BMC Plant Biol. 2012 Dec 17;12:239. doi: 10.1186/1471-2229-12-239 (PMC3548725; doi:10.1186/1471-2229-12-239)
Supplement: Additional file 1 — Table S1. Fresh weight (g), % water content, and total soluble solids (°Brix) in entire tomato fruits of ‘Santorini’ and ‘Ailsa Craig’ at different ripening stages. Ripening stages: IG, Immature Green; MG, Mature Green; B-1, Breaker −1 day; B, Breaker; B + 1, Breaker + 1 day; PK, Pink; R, Red. Results are represented as mean measurements in 10 tomato fruits ± SD. [file 1471-2229-12-239-S1.pdf]

Additional file 1 – Supplemental Table 1 .pdf - Fresh weight (g), % water content, and total soluble solids (°Brix) in entire tomato fruits of 'Santorini' and 'Ailsa Craig' at different ripening stages.

Ripening stages: IG, Immature Green; MG, Mature Green; B-1, Breaker -1 day; B, Breaker; B+1, Breaker + 1 day; PK, Pink; R, Red. Results are represented as mean measurements in 10 tomato fruits ± SD.

	Fresh weight (g)		Water content (%)		°Brix	
Stage	Santorini	Ailsa Craig	Santorini	Ailsa Craig	Santorini	Ailsa Craig
IG	19.66 ± 2.34	27.51 ± 2.31	89.4 ± 2.65	90.4 ± 2.34	4.53 ± 0.11	4.37 ± 0.13
MG	28.87 ± 4.56	45.99 ± 5.67	91.1 ± 3.76	91.0 ± 3.21	4.43 ± 0.07	4.27 ± 0.07
B-1	27.91 ± 4.23	45.85 ± 5.78	91.6 ± 3.23	92.3 ± 2.13	3.73 ± 0.02	3.90 ± 0.12
Br	27.74 ± 4.87	48.94 ± 4.98	90.0 ± 2.54	89.7 ± 3.04	3.83 ± 0.05	3.91 ± 0.08
B+1	27.21 ± 3.76	48.14 ± 5.87	90.8 ± 3.76	89.9 ± 3.12	3.75 ± 0.05	3.99 ± 0.12
PK	27.17 ± 3.22	45.50 ± 6.02	90.2 ± 2.65	89.7 ± 2.14	3.87 ± 0.04	4.23 ± 0.11
R	28.94 ± 3.44	47.84 ± 4.78	90.3 ± 2.87	90.1 ± 1.76	4.23 ± 0.05	4.30 ± 0.09
