# Supplementary material for: Regulation of fruit ascorbic acid concentrations during ripening in high and low vitamin C tomato cultivars
Source: BMC Plant Biol. 2012 Dec 17;12:239. doi: 10.1186/1471-2229-12-239 (PMC3548725; doi:10.1186/1471-2229-12-239)
Supplement: Additional file 2 — Table S2. Determination of L-ascorbic acid and glutathione during fruit development and ripening. Changes in total ascorbic acid (totAsA), L-ascorbic acid (AsA), total glutathione (totGSH), and glutathione (GSH) concentrations (nmol/gFW) during fruit development and ripening of ‘Santorini’ and ‘Ailsa Craig’ fruits. Ripening stages: IG, Immature Green; MG, Mature Green; B-1, Breaker −1 day; B, Breaker; B + 1, Breaker + 1 day; PK, Pink; R, Red. Results are represented as the mean of three biological replications. Statistical analysis was carried out for each cultivar independently. Means of each cultivar with the same letter are not significantly different (a = 0.05) based on Duncan’s multiple-range-test in SAS. [file 1471-2229-12-239-S2.pdf]

Additional file 2 – Supplemental Table 2 .pdf –Determination of L-ascorbic acid and glutathione during fruit development and ripening.
Changes in total ascorbic acid (totAsA), L-ascorbic acid (AsA), total glutathione (totGSH), and glutathione (GSH) concentrations (nmol/gFW) during fruit development and ripening of 'Santorini' and 'Ailsa Craig' fruits. Ripening stages: IG, Immature Green; MG, Mature Green; B-1, Breaker -1 day; B, Breaker; B+1, Breaker + 1 day; PK, Pink; R, Red. Results are represented as the mean of three biological replications. Statistical analysis was carried out for each cultivar independently. Means of each cultivar with the same letter are not significantly different (a=0.05) based on Duncan's multiple-range-test in SAS.

						Santorini							
Stage	DAP	totAsA		AsA		%DHA		totGSH		GSH		%GSSG	
IG	27	1356.6 ± 67.9	ab	729.6 ± 71.5	bcd	46.3 ± 2.54	a	625.9 ± 42.9	a	373.0 ± 31.9	a	40.4 ± 3.14	a
MG	38	1460.0 ± 34.2	ab	725.4 ± 41.2	cd	47.0 ± 1.70	a	611.6 ± 14.2	ab	413.1 ± 15.4	a	32.5 ± 1.30	bc
B-1	40	1301.6 ± 45.3	b	671.8 ± 24.8	d	47.3 ± 2.30	a	597.3 ± 30.5	abc	392.5 ± 30.5	a	34.2 ± 5.34	ab
B	41	1461.5 ± 25.3	a	784.0 ± 27.1	abc	47.7 ± 3.36	a	544.3 ± 17.2	cd	411.6 ± 21.0	a	24.4 ± 2.11	d
B+1	42	1333.9 ± 25.9	ab	741.8 ± 28.7	bcd	44.4 ± 1.92	ab	511.0 ± 48.6	d	393.7 ± 32.2	a	22.8 ± 4.72	d
PK	46	1348.2 ± 25.8	ab	805.1 ± 44.8	ab	40.3 ± 2.94	b	553.9 ± 33.9	bcd	399.2 ± 12.4	a	27.8 ± 2.83	cd
R	50	1204.8 ± 54.5	c	826.4 ± 32.3	a	29.6 ± 2.91	c	523.8 ± 44.5	d	373.3 ± 35.1	a	28.8 ± 1.37	bc
						Ailsa Craig							
Stage	DAP	totAsA		AsA		%DHA		totGSH		GSH		%GSSG	
IG	27	858.20 ± 50.2	de	406.1 ± 33.6	cd	55.2 ± 4.45	bcd	561.0 ± 32.3	a	280.0 ± 19.8	abc	50.1 ± 0.68	ab
MG	39	813.59 ± 38.8	e	299.4 ± 112	e	63.3 ± 0.90	a	559.1 ± 61.4	a	314.5 ± 27.0	a	43.6 ± 1.84	abc
B-1	41	909.24 ± 57.7	d	391.4 ± 35.1	d	52.3 ± 9.82	abc	566.1 ± 97.6	a	261.1 ± 43.2	bc	53.8 ± 1.71	a
B	42	1244.4 ± 55.8	a	677.4 ± 28.8	a	45.6 ± 1.20	d	419.5 ± 51.5	b	301.2 ± 43.2	ab	27.6 ± 7.58	d
B+1	43	1159.2 ± 38.7	ab	563.4 ± 16.1	b	51.4 ± 1.13	bcd	416.3 ± 42.5	b	236.7 ± 19.5	c	41.3 ± 7.71	abc
PK	47	1182.5 ± 16.9	ab	486.6 ± 13.1	bc	58.8 ± 0.79	abc	489.9 ± 65.6	ab	293.9 ± 18.3	ab	39.4 ± 6.02	bcd
R	52	1005.2 ± 18.5	c	493.5 ± 17.3	bc	51.2 ± 3.55	cd	405.9 ± 28.3	b	274.8 ± 28.7	abc	32.3 ± 4.01	cd
