# Supplementary material for: Regulation of fruit ascorbic acid concentrations during ripening in high and low vitamin C tomato cultivars
Source: BMC Plant Biol. 2012 Dec 17;12:239. doi: 10.1186/1471-2229-12-239 (PMC3548725; doi:10.1186/1471-2229-12-239)
Supplement: Additional file 3 — Table S3. Non-radiolabelled feeding experiments with AsA precursor substrates. The effect of incubating with D-glucose (D-Glc), D-mannose (D-Man), L-galactose (L-Gal), and L-galactono-lactone (L-GaL) from the L-galactose pathway, L-gulono-lactone (L-GuL), D-glucurono-lactone (D-GlcUL), and myo-inositol (MI) from the alternative biosynthetic pathways, or AsA itself and DHA for 24 hours on total AsA (totAsA) concentrations (mmol/gFW) of immature green (IG), mature green (MG), and red fruit discs of ‘Santorini’ and ‘Ailsa Craig’. Results represent means of three replications ± SD, and asterisks indicate values that are significantly different from those of the control for 24 hours (t-test, P < 0.05). [file 1471-2229-12-239-S3.pdf]

Additional file 3 – Supplemental Table 3 .pdf - Non-radiolabelled feeding experiments with AsA precursor substrates.
The effect of incubating with D-glucose (D-Glc), D-mannose (D-Man), L-galactose (L-Gal), and L-galactono-lactone (L-GaL) from the L-galactose pathway, L-gulono-lactone (L-GuL), D-glucurono-lactone (D-GlcUL), and *myo* -inositol (MI) from the alternative biosynthetic pathways, or AsA itself and DHA for 24 hours on total AsA (totAsA) concentrations (mmol/gFW) of immature green, mature green, and red fruit discs of 'Santorini' and 'Ailsa Craig'. Results represent means of three replications ± SD, and asterisks indicate values that are significantly different from those of the control for 24 hours (t-test, P<0.05).

			Immature Green			Mature Green			Red		
Variety	Substrate	Time	TotAA		Relative rate	TotAA		Relative rate	TotAA		Relative rate
Santorini	control	0h	0.95 ± 0.06			0.59 ± 0.11			1.00 ± 0.06		
	control	24h	0.54 ± 0.10			0.75 ± 0.11			0.51 ± 0.04		
	D-Glc		0.49 ± 0.06		0.92 ± 0.11	0.61 ± 0.04		0.82 ± 0.05	0.51 ± 0.09		1.00 ± 0.18
	D-man		0.54 ± 0.08		1.01 ± 0.16	0.70 ± 0.09		0.94 ± 0.12	0.56 ± 0.02		1.08 ± 0.04
	L-Gal		1.17 ± 0.40	*	2.17 ± 0.79	0.99 ± 0.00	*	1.32 ± 0.00	0.63 ± 0.13		1.23 ± 0.25
	L-GaL		0.75 ± 0.06		1.39 ± 0.10	0.96 ± 0.15	*	1.28 ± 0.20	0.71 ± 0.11	*	1.37 ± 0.22
	L-GuL		0.34 ± 0.02		0.64 ± 0.04	0.66 ± 0.06		0.88 ± 0.08	0.45 ± 0.09		0.87 ± 0.17
	D-GlcUL		0.37 ± 0.10		0.68 ± 0.20	0.60 ± 0.01		0.80 ± 0.14	0.57 ± 0.04		1.10 ± 0.07
	MI		0.43 ± 0.07		0.80 ± 0.14	0.65 ± 0.03		0.75 ± 0.05	0.54 ± 0.14		1.05 ± 0.28
	control		0.54 ± 0.10			0.75 ± 0.11			0.51 ± 0.04		
	AsA		1.23 ± 0.20	*	2.28 ± 0.37	2.34 ± 0.29	*	3.13 ± 0.39	2.87 ± 0.64	*	5.59 ± 1.25
	DHA		2.44 ± 0.50	*	4.52 ± 0.92	2.38 ± 0.20	*	3.19 ± 0.27	0.90 ± 0.41		1.75 ± 0.80
Ailsa Craig	control	0h	0.50 ± 0.10			0.35 ± 0.06			0.56 ± 0.09		
	control	24h	0.26 ± 0.14			0.30 ± 0.02			0.23 ± 0.03		
	D-Glc		0.18 ± 0.09		0.70 ± 0.36	0.37 ± 0.13		1.24 ± 0.44	0.24 ± 0.08		1.06 ± 0.37
	D-man		0.20 ± 0.07		0.78 ± 0.28	0.39 ± 0.09		1.30 ± 0.30	0.46 ± 0.05	*	2.03 ± 0.19
	L-Gal		0.73 ± 0.05	*	2.79 ± 0.21	0.73 ± 0.06	*	2.46 ± 0.19	0.46 ± 0.04	*	2.01 ± 0.19
	L-GaL		0.63 ± 0.08	*	2.43 ± 0.34	0.85 ± 0.07	*	2.87 ± 0.25	0.44 ± 0.01	*	1.92 ± 0.05
	L-GuL		0.14 ± 0.03		0.53 ± 0.13	0.54 ± 0.06	*	1.80 ± 0.19	0.34 ± 0.05		1.51 ± 0.20
	D-GlcUL		0.18 ± 0.02		0.70 ± 0.07	0.35 ± 0.09		1.18 ± 0.30	0.25 ± 0.07		1.11 ± 0.32
	MI		0.16 ± 0.05		0.61 ± 0.19	0.35 ± 0.07		1.12 ± 0.14	0.34 ± 0.03	*	1.49 ± 0.15
	control		0.26 ± 0.14			0.30 ± 0.02			0.23 ± 0.03		
	AsA		1.54 ± 0.08	*	5.93 ± 0.32	1.59 ± 0.14	*	5.35 ± 0.46	1.39 ± 0.15	*	6.09 ± 0.67
	DHA		2.17 ± 0.25	*	8.36 ± 0.95	2.03 ± 0.47	*	6.83 ± 1.58	1.01 ± 0.01	*	4.43 ± 0.05
