# Supplementary material for: Regulation of fruit ascorbic acid concentrations during ripening in high and low vitamin C tomato cultivars
Source: BMC Plant Biol. 2012 Dec 17;12:239. doi: 10.1186/1471-2229-12-239 (PMC3548725; doi:10.1186/1471-2229-12-239)
Supplement: Additional file 4 — Table S4. Correlations between ascorbic acid and enzyme activities of ‘Santorini’ and ‘Ailsa Craig’ fruits during ripening. Pearson correlation coefficients between ascorbic acid (AsA), total ascorbic acid (totAsA), % dehydroascorbate (% DHA) and enzyme activities of ascorbate peroxidise (APX), monodehydroascorbate reductase (MDHAR), dehydroascorbate reductase (DHAR), glutathione reductase (GR), catalase (CAT), superoxide dismutase (SOD), and peroxidase (POX) in ‘Santorini’ and ‘Ailsa Craig’ fruits during ripening. *P < 0.05, **P < 0.01, ***P < 0.001, ****P < 0.0001, n.s. not significant. [file 1471-2229-12-239-S4.pdf]

Additional file 4 – Supplemental Table 4 .pdf – Correlations between ascorbic acid and enzyme activities of 'Santorini' and 'Ailsa Craig' fruits during ripening.

Pearson correlation coefficients between ascorbic acid (AsA), total ascorbic acid (totAsA), % dehydroascorbate (% DHA) and enzyme activities of ascorbate peroxidise (APX), monodehydroascorbate reductase (MDHAR), dehydroascorbate reductase (DHAR), glutathione reductase (GR), catalase (CAT), superoxide dismutase (SOD), and peroxidase (POX) in 'Santorini' and 'Ailsa Craig' fruits during ripening. *P<0.05, **P<0.01, ***P<0.001, ****P<0.0001, n.s. not significant.

		Santorini			Ailsa Craig		
		AsA	totAsA	%DHA	AsA	totAsA	%DHA
Enzyme activity	APX	0.705***	-0.453*	-0.819****	-0.539*	-0.592*	n.s.
	MDHAR	0.669***	n.s.	-0.566**	n.s.	n.s.	n.s.
	DHAR	n.s.	n.s.	n.s.	n.s.	n.s.	n.s.
	GR	n.s.	n.s.	n.s.	n.s.	n.s.	n.s.
	CAT	n.s.	n.s.	-0.465*	0.756****	0.777****	-0.517*
	SOD	n.s.	n.s.	n.s.	0.461*	n.s.	-0.534*
	POX	0.46*	n.s.	n.s.	n.s.	n.s.	n.s.
