# Supplementary material for: Regulation of fruit ascorbic acid concentrations during ripening in high and low vitamin C tomato cultivars
Source: BMC Plant Biol. 2012 Dec 17;12:239. doi: 10.1186/1471-2229-12-239 (PMC3548725; doi:10.1186/1471-2229-12-239)
Supplement: Additional file 5 — Table S5. List of primer sequences of the reference genes tested for stability across ripening stages of ‘Ailsa Craig’ fruits. The stability values for the reference genes were evaluated in ‘Ailsa Craig’ fruits throughout ripening using the NormFinder software. [file 1471-2229-12-239-S5.pdf]

Additional file 5 – Supplemental Table 5 .pdf - List of primer sequences of the reference genes tested for stability across ripening stages of 'Ailsa Craig' fruits.

The stability values for the reference genes were evaluated in 'Ailsa Craig' fruits throughout ripening using the NormFinder software.

Gene	Gene Description	F-primer (5'-3')	R-primer (5'-3')	Reference	Stability values
EF1a	Elongation factor 1 alpha	TGATCAAGCCTGGTATGGTTGT	CTGGGTCATCCTTGGAGTT	Badejo et al, 2011	0.737
PP2Acs	Protein phosphatase 2A catalytic subunit	CGATGTGTGATCTCCTATGGTC	AAGCTGATGGGCTCTAGAAATC	Lovdal and Lillo, 2009	0.369
RPL2	Ribosomal protein L2	GTCATCCTTTCAGGTACAAGCA	CGTTACAAACAACAGCTCCTTC	Lovdal and Lillo, 2009	0.519
SAND	SAND family protein	TTGCTTGGAGGAACAGACG	GCAAACAGAACCCCTGAATC	Rodriguez et al, 2008	0.423
CAC	Clathrin adaptor complexes medium subunit	CCTCCGTTGTGATGTAACTGG	ATTGGTGGAAAGTAACATCATCG	Rodriguez et al, 2008	0.344
ACT1	Actin	TGTCCCTATTTACGAGGGTTATGC	CAGTTAAATCACGACCAGCAAGAT	Girardi et al, 2009	0.568
ACT2	Actin	GAAATAGCATAAGATGGCAGACG	ATACCCACCATCACACCAGTAT	Lovdal and Lillo, 2009	1.034
