# Supplementary material for: Regulation of fruit ascorbic acid concentrations during ripening in high and low vitamin C tomato cultivars
Source: BMC Plant Biol. 2012 Dec 17;12:239. doi: 10.1186/1471-2229-12-239 (PMC3548725; doi:10.1186/1471-2229-12-239)
Supplement: Additional file 6 — Table S6. Correlations between ascorbic acid and gene expression of ‘Ailsa Craig’ fruits around the breaker stage. Pearson correlation coefficients of the expression of AsA-related genes from biosynthetic and recycling pathways (listed in Table 2) measured at one day before breaker, breaker and one day after breaker stages, with ascorbic acid (AsA), and total ascorbic acid (totAsA) measured at the breaker, one day after breaker and two days after breaker stages i.e. assuming a delay of one day between gene expression and changes in fruit AsA concentrations. *P < 0.05, **P < 0.01, ***P < 0.001, ****P < 0.0001, n.s. not significant. [file 1471-2229-12-239-S6.pdf]

Additional file 6 – Supplemental Table 6 .pdf – Correlations between ascorbic acid and gene expression of 'Ailsa Craig' fruits around the breaker stage.

Pearson correlation coefficients of the expression of AsA-related genes from biosynthetic and recycling pathways (listed in Table 2) measured at one day before breaker, breaker and one day after breaker stages, with ascorbic acid (AsA), and total ascorbic acid (totAsA) measured at the breaker, one day after breaker and two days after breaker stages i.e. assuming a delay of one day between gene expression and changes in fruit AsA concentrations. *P<0.05, **P<0.01, ***P<0.001, ****P<0.0001, n.s. not significant.

		AsA	totAsA
Gene expression	*SlGMP1*	n.s.	n.s.
	SlGMP2	0.736*	n.s.
	SlGMP3	n.s.	n.s.
	SlGME1	n.s.	n.s.
	SlGME2	n.s.	n.s.
	SlGGP1	0.963****	0.929***
	SlGGP2	0.725*	n.s.
	SlGPP1	n.s.	n.s.
	SlGPP2	n.s.	n.s.
	SlGalDH	n.s.	n.s.
	SlGLDH	n.s.	n.s.
	SlMDHAR1	0.849***	0.850***
	SlMDHAR2	n.s.	n.s.
	SlMDHAR3	0.898***	0.847***
	SlDHAR1	n.s.	n.s.
	SlDHAR2	n.s.	n.s.
