# Supplementary material for: Regulation of fruit ascorbic acid concentrations during ripening in high and low vitamin C tomato cultivars
Source: BMC Plant Biol. 2012 Dec 17;12:239. doi: 10.1186/1471-2229-12-239 (PMC3548725; doi:10.1186/1471-2229-12-239)
Supplement: Additional file 7 — Table S7. Composition of the nutrient solution used in the hydroponically-grown tomatoes. [file 1471-2229-12-239-S7.pdf]

**Additional file 7 – Supplemental Table 7 .pdf – Composition of the nutrient solution used in the hydroponically-grown tomatoes.**

| **Nutrient** | **Concentration** |
|---|---|
| Macronutrients | mmol/L |
| $K^+$ | 9.1 |
| $Ca^{++}$ | 6.5 |
| $Mg^{++}$ | 2.0 |
| $NO_3^-$ | 16.3 |
| $H_2PO_4^-$ | 2.0 |
| $SO_4^-$ | 3.9 |
| Micronutrients | µmol/L |
| $ZnSO_4.7H_2O$ | 5.61 |
| $CuSO_4.5H_2O$ | 1.62 |
| $MnSO_4.H_2O$ | 76.9 |
| $H_3BO_3$ | 20.1 |
| $(NH_4)_6Mo_7O_2.4H_2O$ | 1.15 |
